# Shared senescence-associated gene networks in PCOS and T2DM: biomarker identification and functional validation

**DOI:** 10.3389/fendo.2025.1652178

**Published:** 2025-09-25

**Authors:** Mingyue Kong, Kun Lou, Dan Liu, Ziqi Dai, Tianjiao Li, Yihan Yang, Xuesong Zhang, Xin Chen

**Affiliations:** ^1^ College of Traditional Chinese Medicine, Changchun University of Chinese Medicine, Changchun, Jilin, China; ^2^ Changchun Orthopedics Hospital, Changchun, Jilin, China; ^3^ Affiliated Hospital of Changchun University of Chinese Medicine, Changchun, Jilin, China; ^4^ Changchun University of Chinese Medicine, Changchun, Jilin, China; ^5^ Gynecology Department, Affiliated Hospital of Changchun University of Chinese Medicine, Changchun, China

**Keywords:** bioinformatics analysis, hub genes, molecular interaction networks, senescence-associated secretory phenotype, polycystic ovary syndrome, type 2 diabetes

## Abstract

**Background:**

Polycystic ovary syndrome (PCOS) and type 2 diabetes mellitus (T2DM) are two prevalent and interrelated disorders that pose an increasingly significant global health burden. Cellular senescence may represent a pivotal process driving the progression of both conditions. Senescent cells, through the senescence-associated secretory phenotype (SASP), can induce chronic inflammation, which is highly likely to exacerbate the pathological progression of PCOS and T2DM. However, the molecular pathways linking cellular senescence to PCOS and T2DM have not yet been systematically elucidated.

**Methods:**

The transcriptome datasets of PCOS (GSE54248) and T2DM (GSE23561) were obtained from the GEO database, and differentially expressed genes (DEGs) were screened using the limma package. Age-related DEGs (ARDEGs) were obtained by intersecting DEGs with age-related genes, and the protein-protein interaction (PPI) network was constructed based on the STRING database. Hub genes with diagnostic value were determined via the Wilcoxon rank sum test and receiver operating characteristic (ROC) curve. CIBERSORT was used to analyze the infiltration characteristics of immune cells, and the functions of the hub gene were analyzed by gene set enrichment analysis (GSEA). Single-cell sequencing was used to locate gene expression patterns, and qRT–PCR was used to verify the expression of candidate genes in clinical samples.

**Results:**

80 DEGs between PCOS and T2DM samples were obtained, and 15 ARDEGs were identified. Gene Ontology (GO) and Kyoto Encyclopedia of Genes and Genomes (KEGG) analysis showed that they were related to inflammatory response and immune response, and were involved in specific functions and pathways. Four hub genes were identified: TUBA4A, RTN1, G6PD, and HP. qRT–PCR experimental results showed that HP, G6PD, TUBA4A, and RTN1 were highly expressed in the peripheral blood of PCOS and T2DM patients, compared to healthy people.

**Discussion:**

This study revealed the potential connections between PCOS, T2DM, and aging-related molecular networks and signaling pathways and discovered multiple potential therapeutic targets. It provides new intervention directions for clinicians, especially based on aging mechanisms.

## 1 Introduction

As the most prevalent endocrine-metabolic disorder among women of reproductive age, polycystic ovary syndrome (PCOS) is characterized by a dual clinical profile involving both reproductive and metabolic abnormalities (1). Reproductive manifestations of PCOS include menstrual irregularities (such as amenorrhea and oligo-ovulation) (2), infertility (3), and polycystic ovarian morphology (4). Metabolic abnormalities are characterized by obesity (5), insulin resistance (IR) (6), and hyperandrogenism-related features such as hirsutism (7), acne (8), etc. Type 2 diabetes mellitus (T2DM), a common metabolic disorder with high global prevalence, predominantly features pancreatic β-cell dysfunction and IR in peripheral target organs (9). In recent years, increasing evidence has indicated a significant comorbidity trend between PCOS and T2DM at both the clinical and molecular levels: 1) regarding genetic susceptibility, genome-wide association studies (GWAS) have identified multiple shared genetic risk loci (10); 2) In terms of pathophysiology, hyperinsulinemia may synergistically potentiate the action of luteinizing hormone (LH), thereby stimulating excessive androgen secretion from ovarian theca-interstitial cells (11); 3) As for epidemiology, women with PCOS have a 4- to 8-fold higher risk of developing T2DM compared with the general population (12). Nevertheless, the core molecular mechanisms underpinning the coordinated progression of these two conditions remain fundamentally contentious. Emerging evidence suggests that PCOS and cellular senescence may share pathogenic mechanisms, including oxidative stress, chronic low-grade inflammation, and immune dysregulation (13). Moreover, accelerated ovarian granulosa cell senescence in PCOS and an increased proportion of senescent cells in adipose tissue in T2DM have been consistently reported (14, 15).

In light of these observations, cellular senescence may represent a common pathological link between PCOS and T2DM. Several experimental findings lend support to this hypothesis (1): In a bisphenol A-induced rat model of PCOS, expression of the cellular senescence marker p16^INK4A^ in ovarian tissue was elevated 2.3-fold (p < 0.01), and the activity of senescence-associated β-galactosidase (SA-β-gal) exhibited a positive correlation with the rate of follicular atresia (r=0.78) (16) (2); Experimental studies have demonstrated that miR-424-5p is downregulated in granulosa cells and exosomes derived from the follicular fluid of patients with PCOS, and that this downregulation promotes cellular senescence by inducing senescence phenotypes in human granulosa cell lines (COV434 and KGN) (17) (3). In patients with T2DM, the proportion of senescent cells in adipose tissue was 40% higher than in healthy controls, and senescence-associated secretory phenotype (SASP) factors such as IL-6 and CCL2 secreted by these cells may exacerbate IR via the JAK/STAT signaling pathway (15, 18) (4); Preclinical studies have demonstrated that selective clearance of senescent cells can improve islet function by 35% in diabetic mice, providing theoretical support for senescence-targeted therapies (19).

These findings suggest that cellular senescence may represent a common pathological link between PCOS and T2DM. Nevertheless, the senescence-associated molecular networks shared across different tissues remain unclear. In this context, the present study focused on elucidating both disease-specific and shared gene molecular signatures and their interrelationships, to integrate multi-omics data to identify cellular senescence-related molecular networks common to PCOS and T2DM, to validate the cross-tissue diagnostic value of hub genes, and to clarify the central role of cellular senescence in the comorbidity mechanism.

## 2 Methods

### 2.1 Data collection and processing

Microarray datasets for PCOS and T2DM were retrieved from the Gene Expression Omnibus (GEO) database (http://www.ncbi.nlm.nih.gov/geo) via the “GEOquery” R package (version 2.26.1) (20). The GSE54248 dataset comprises total RNA extracted from peripheral blood samples of four patients with PCOS and four healthy controls. For T2DM, the GSE23561 dataset, containing peripheral blood samples from nine healthy individuals and eight patients with T2DM, was selected for analysis. Furthermore, to evaluate the diagnostic accuracy of the identified hub genes as biomarkers for PCOS and T2DM, granulosa cell datasets from PCOS patients and peripheral blood datasets from T2DM patients were obtained from the GEO database. Batch effects were removed using “sva” in R 3.52.0, resulting in the construction of two integrated datasets: the PCOS granulosa cell dataset (PCOS_GC_DATASET), comprising GSE106724, GSE114419, and GSE137684 (19 PCOS and 11 control samples); and the T2DM peripheral blood mononuclear cell dataset (T2DM_PBMC_DATASET), comprising GSE156993, GSE15932, and GSE9006 (32 T2DM and 38 control samples) (**Table 1**). The integrated GEO datasets were subsequently standardized and normalized using “limma” in R 3.60.2 (21), and probe annotation was performed. Principal component analysis (PCA) was conducted both before and after batch effect removal to assess the effectiveness of the adjustment.

**Table 1:** Detailed information on the two datasets.

GEO No.	Database	Data type	Description
GSE54248	GEO	mRNA	Peripheral blood samples from 4 PCOS sufferers and 4 healthy controls
GSE23561	GEO	mRNA	Peripheral blood samples from 8 T2DM patients and 9 healthy controls
PCOS_GC_DATASET	GEO	mRNA	Granulosa cell samples from 19 PCOS sufferers and 11 healthy controls
T2DM_PBMC_DATASET	GEO	mRNA	Peripheral blood samples from 32 T2DM patients and 38 healthy controls

### 2.2 Identification of age-related differentially expressed genes

DEGs from each dataset were found via the R package “limma,”with the criteria set as p < 0.05 and |logFC| > 0.585. Variance between samples was corrected using empirical Bayes moderation (eBayes function). The resulting DEGs were visualized using volcano plots and heatmaps generated by the R packages “ggplot2” (22)and “pheatmap (23). Common DEGs across the GSE54248 and GSE23561 datasets were detected and visually presented via the R package “ggvenn.” (24). ARDEGs were sourced from Msigdb (https://www.gsea-msigdb.org/gsea/msigdb), CellAge (https://genomics.senescence.info/cells/), and Aging Atlas (https://ngdc.cncb.ac.cn/aging/index). The intersection of common DEGs and ARDEGs was determined and visualized using the R package “ggvenn.”

### 2.3 Enrichment analysis of DEGs

Kyoto Encyclopedia of Genes and Genomes (KEGG) pathway and Gene Ontology (GO) enrichment analyses, involving Biological Processes (BP), Cellular Components (CC), and Molecular Functions (MF), were performed. Enrichment analysis was conducted using the R package “ClusterProfiler” via hypergeometric testing (25), with a p-value < 0.05 as the threshold, to comprehensively obtain functional annotations of the DEGs.

### 2.4 Protein-protein interaction network construction and hub gene analysis

To gain an in-depth understanding of the proteins encoded by DEGs and their interactions, interacting genes were retrieved from the STRING database (https://string-db.org/) to construct a protein-protein interaction (PPI) network (26). The network was visualized via Cytoscape 3.8.0 (27), and the CytoHubba plugin was employed to identify hub genes within the network. Candidate hub genes were defined as the top ten genes based on a comprehensive ranking of node degree (threshold ≥ 2), betweenness centrality, and Maximal Clique Centrality (28).

### 2.5 Single-gene gene set enrichment analysis

This study calculated the correlations between four target genes and other genes, and based on the strength of these correlations, relevant gene sets were identified. GSEA was carried out on the key gene sets using the ClusterProfiler package (v4.10.1).

### 2.6 Immunoinfiltration analysis

The CIBERSORT R package (29)was used to estimate the expression of 20 immune cell types in each sample from patients with PCOS. Subsequently, Spearman correlation analysis was conducted to assess the relationship between immune cells and hub genes with a significance threshold of p < 0.05.

### 2.7 Validation of differential expression of hub genes and receiver operating characteristic (ROC) curve analysis

Comparative plots based on hub gene expression levels were constructed to further explore the differential expression of hub genes between the PCOS and T2DM groups in the integrated GEO datasets. Statistical analysis was performed using the Wilcoxon rank-sum test (a nonparametric test suitable for small sample sizes and non-normally distributed data). Subsequently, ROC curves for hub genes were generated using the R package pROC (30), and the area under the curve (AUC) was calculated to assess the diagnostic performance of hub gene expression for the two diseases.

### 2.8 Single-cell transcriptome data

To investigate the expression patterns of key genes in peripheral blood, the T2DM dataset (GSE28040) was downloaded, and single-cell transcriptomic data were processed using the Seurat package. After rigorous quality control, high-quality cells with 200- 2,500 detected genes and less than 5% mitochondrial gene content (percent.mt) were retained. The data were then normalized and variance-stabilized. Subsequent analyses were performed using the top 2,000 most variable genes from each sample. To eliminate batch effects, the Harmony algorithm was used for data integration, followed by gene expression scaling using the ScaleData function. Dimensionality reduction was performed via PCA with a resolution parameter of 0.8. Cell clustering was further conducted using the FindNeighbors and FindClusters functions. Cell type annotation was automatically performed via the SingleR tool.

### 2.9 Real-time quantitative polymerase chain reaction

To confirm the accuracy of the foregoing hub genes, a quantitative reverse transcription polymerase chain reaction (qRT-PCR) was performed on the four selected genes. Nine subjects were recruited for this validation study, consisting of three healthy controls, three PCOS patients, and three T2DM patients. Peripheral blood samples were gathered from all subjects via venipuncture. Total RNA was extracted via TRIzol (Invitrogen). Reverse transcription was enabled by the HiScript III RT SuperMix (+gDNA wiper) kit (Cat. No. 11201ES08, Yisheng Biotechnology Co., Ltd., Shanghai). qRT–PCR was performed through the ChamQ Universal SYBR qRT–PCR Master Mix (Vazume) based on the LightCycler^®^ 480 II real-time PCR system (Roche), with GAPDH serving as the internal reference gene. Each sample was run in triplicate. The relative expression levels were calculated using the 2-ΔΔCt approach. Normality was assessed using the Shapiro-Wilk test (p > 0.05 considered normally distributed). Intergroup comparisons were conducted using one-way analysis of variance (ANOVA) for normally distributed data or the Kruskal-Wallis test for non-normally distributed data, followed by Tukey’s HSD *post hoc* test. Data are presented as mean ± standard deviation (SD), and statistical significance was defined as p < 0.05. This experiment was supported by the Ethics Committee of the Affiliated Hospital of Changchun University of Traditional Chinese Medicine, ethical review number: CCZYFYKYLL2025.

### 2.10 Statistical analysis

The foregoing bioinformatics analyses and R packages were enabled by R (v4.0.3). Pearson correlation analysis was employed for correlation clarification, and the Wilcoxon rank-sum test was applied to assess the significance across groups. P<0.05 suggested statistical significance.

## 3 Results

**Figure 1:**
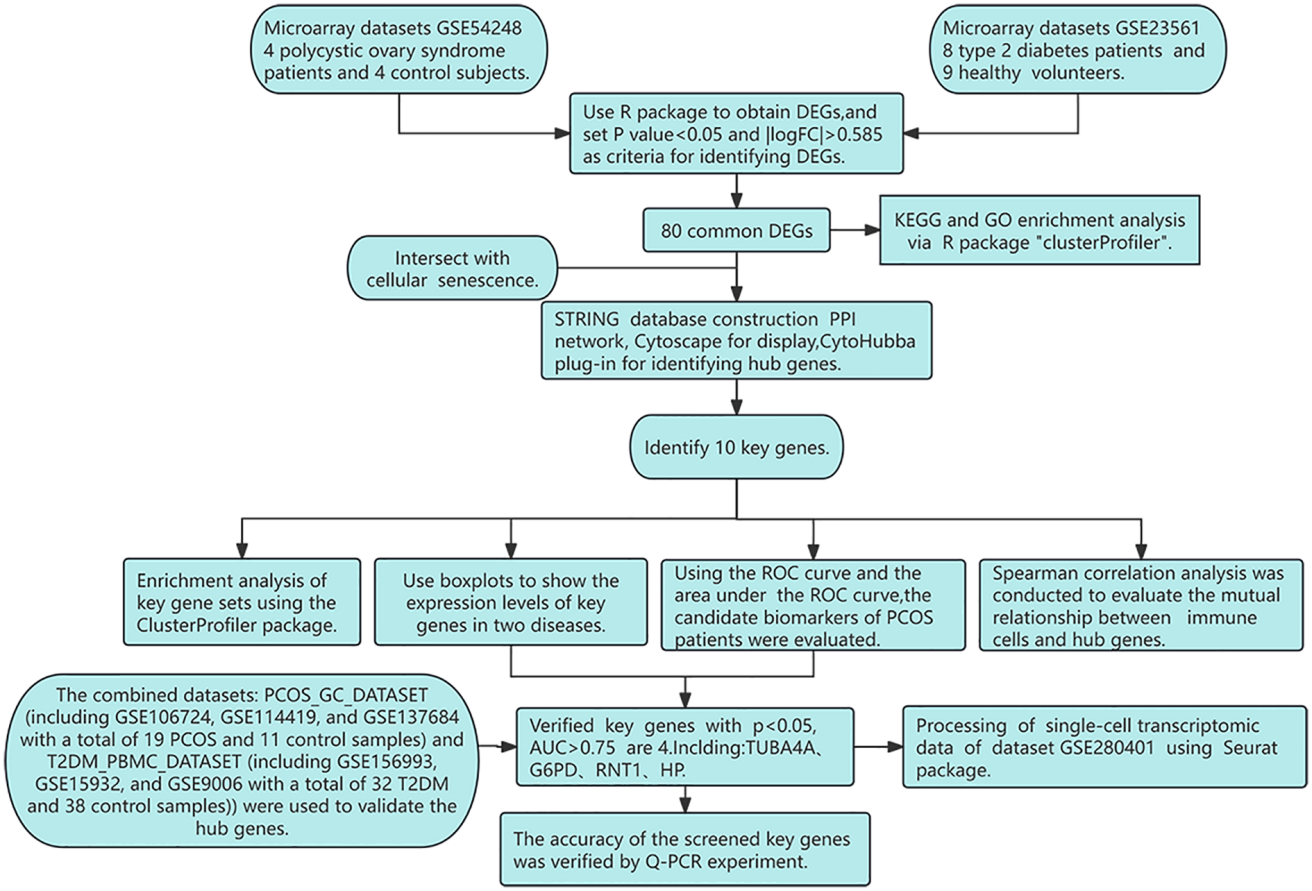
Research design flowchart.

The workflow for identifying common DEGs between PCOS and T2DM is presented in **Figure 1**.

### 3.1 Identification of DEGs in PCOS and T2DM

**Figure 2:**
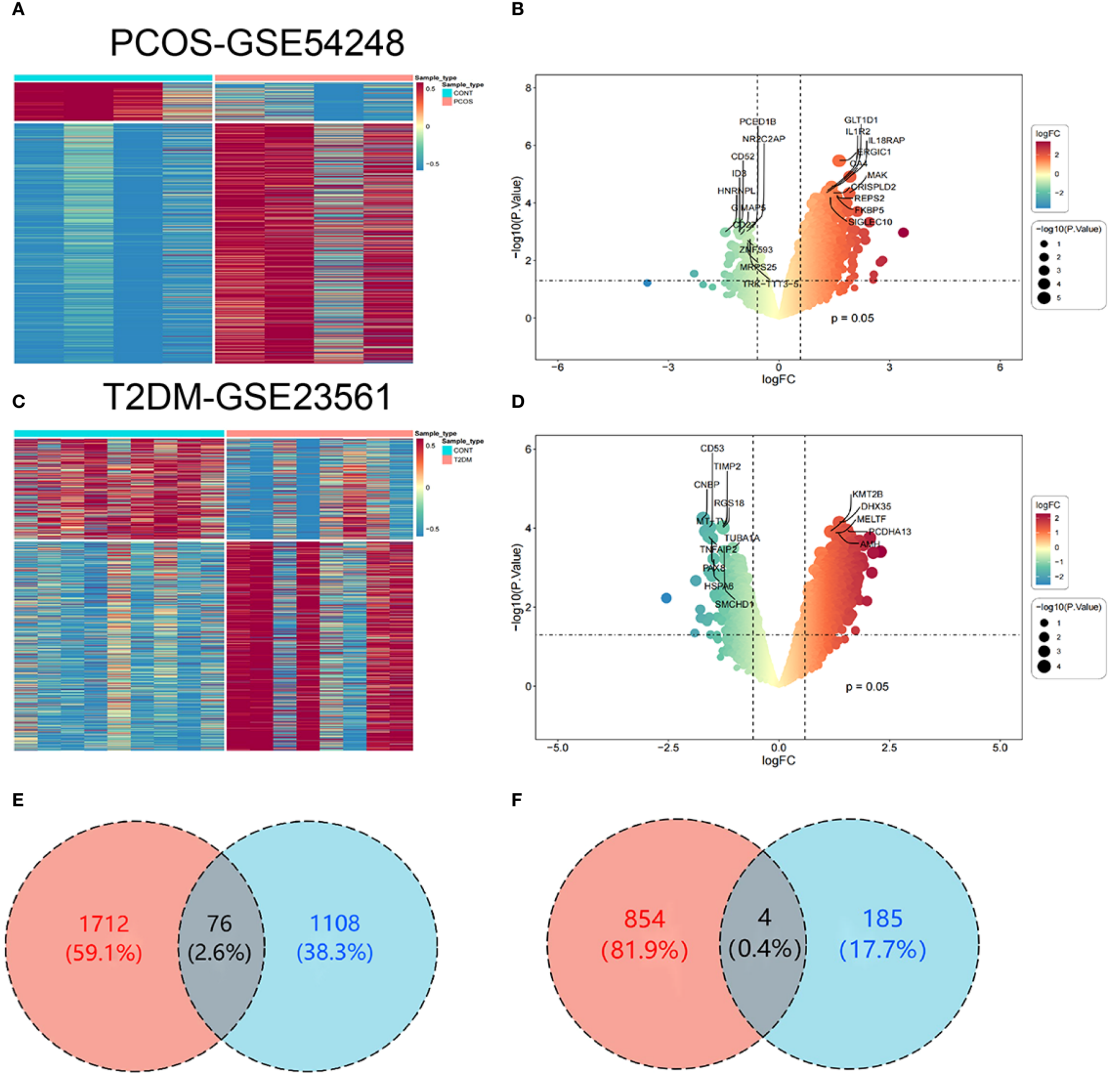
Identification of DEGs. **(A)** Heatmap of DEGs in the PCOS dataset (GSE54248). **(B)** Volcano plot depicting the distribution of DEGs in the PCOS dataset. **(C)** Heatmap of DEGs in the T2DM dataset (GSE23561). **(D)** Volcano plot showing the DEG distribution in the T2DM dataset. **(E)** Venn diagram of upregulated DEGs common to both PCOS and T2DM. **(F)** Venn diagram of downregulated DEGs common to both PCOS and T2DM.

To investigate the interactions and cross-talk between PCOS and T2DM, analysis was executed on the GSE54248 and GSE23561 datasets from the GEO. DEGs were identified with |log2 FC| > 0.585 and p-value < 0.05. The GSE54248 dataset was used to identify DEGs in PCOS patients, yielding 1,373 DEGs (**Figure 2A**). The volcano plot illustrates the identified DEGs, comprising 1,184 upregulated genes and 189 downregulated genes (**Figure 2B**). The GSE23561 dataset revealed 2,646 DEGs (**Figure 2C**), of which 1,788 were upregulated and 858 were downregulated (**Figure 2D**). Among these DEGs, 76 were commonly upregulated (**Figure 2E**) and 4 were commonly downregulated (**Figure 2F**) following the intersection of the DEGs from both PCOS and T2DM datasets (**Supplementary Table 1**).

### 3.2 Integration of PCOS and T2DM datasets

**Figure 3:**
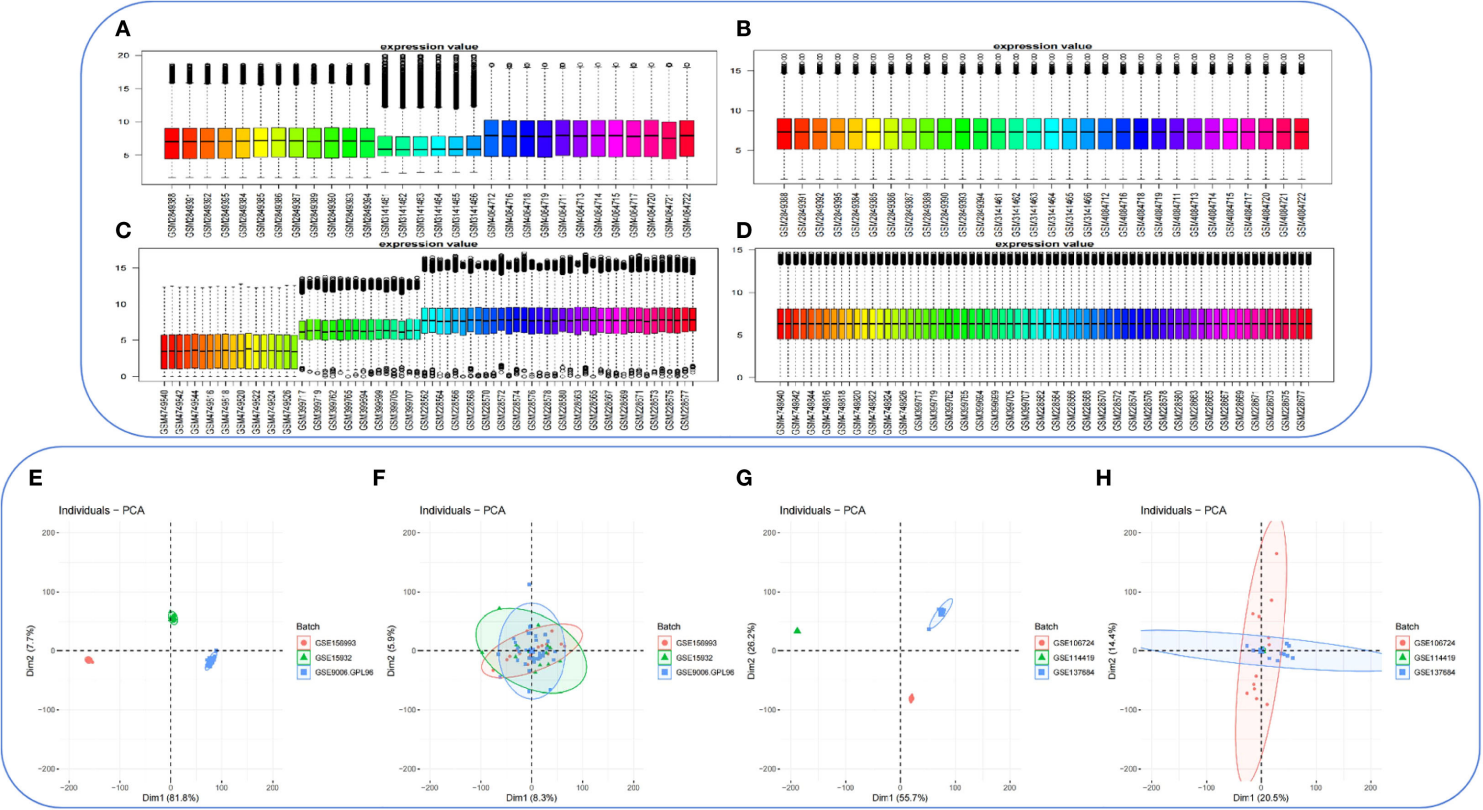
Batch effect removal in the PCOS_GC_DATASET and T2DM_PBMC_DATASET. **(A)** Boxplot depicting the distribution of the combined GEO dataset PCOS_GC_DATASET prior to batch effect correction. **(B)** Boxplot depicting the distribution of the integrated GEO dataset PCOS_GC_DATASET following batch effect correction. **(C)** Boxplot depicting the distribution of the combined GEO dataset T2DM_PBMC_DATASET prior to batch effect correction. **(D)** Boxplot depicting the distribution of the integrated GEO dataset T2DM_PBMC_DATASET following batch effect correction. **(E)** Principal component analysis (PCA) of the PCOS_GC_DATASET prior to batch effect correction. **(F)** PCA of the PCOS_GC_DATASET following batch effect correction. **(G)** PCA of the T2DM_PBMC_DATASET prior to batch effect correction. **(H)**PCA of the T2DM_PBMC_DATASET following batch effect correction. PCA, principal component analysis.

The R package sva was applied to the PCOS_GC_DATASET and T2DM_PBMC_DATASET to remove batch effects and generate a combined GEO dataset. Violin plots (**Figures 3A–D**) and PCA (**Figures 3E-H**) were used to compare the datasets before and after batch correction. Following batch effect adjustment, the batch-related variability within the datasets was successfully eliminated.

### 3.3 Functional enrichment analysis of DEGs

To elucidate the possible molecular pathways contributing to T2DM susceptibility in PCOS patients, GO enrichment and KEGG pathway analyses were executed on the 80 shared DEGs.

**Figure 4:**
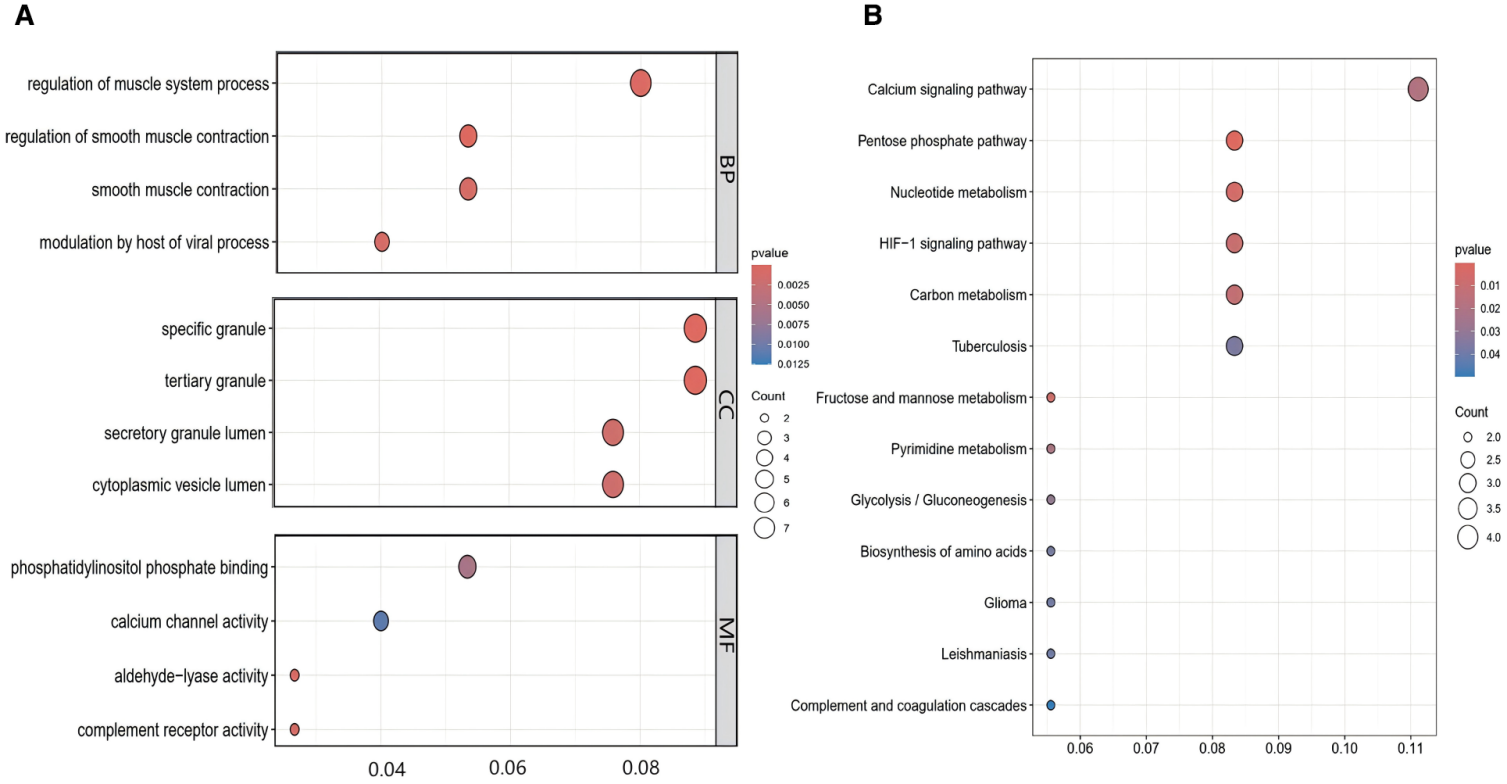
GO Enrichment and KEGG pathway analysis of co-expressed genes between PCOS and T2DM. **(A)** GO enrichment analysis of the intersecting genes. **(B)** KEGG pathway enrichment analysis of the intersecting genes.

GO enrichment analysis demonstrated significant pathways enriched in various categories (p-value < 0.05), including BP, CC, and MF. According to the GO analysis, they were mainly located in cellular granules and their components, involved in immune and inflammatory responses, regulation of smooth muscle contraction, and calcium ion transport signaling pathways. The functions associated with these genes included regulation of calcium channel activity, glycosyltransferase activity, and phosphatidylinositol-3-phosphate binding (**Figure 4A**).KEGG pathway analysis highlighted significant enrichment of these genes in pathways related to carbohydrate metabolism, nucleotide metabolism, HIF-1 signaling, and calcium signaling (**Figure 4B**). Therefore, immune activation and inflammatory responses are pivotal in the progression of both PCOS and T2DM.

### 3.4 PPI network analysis and identification of hub genes

**Figure 5:**
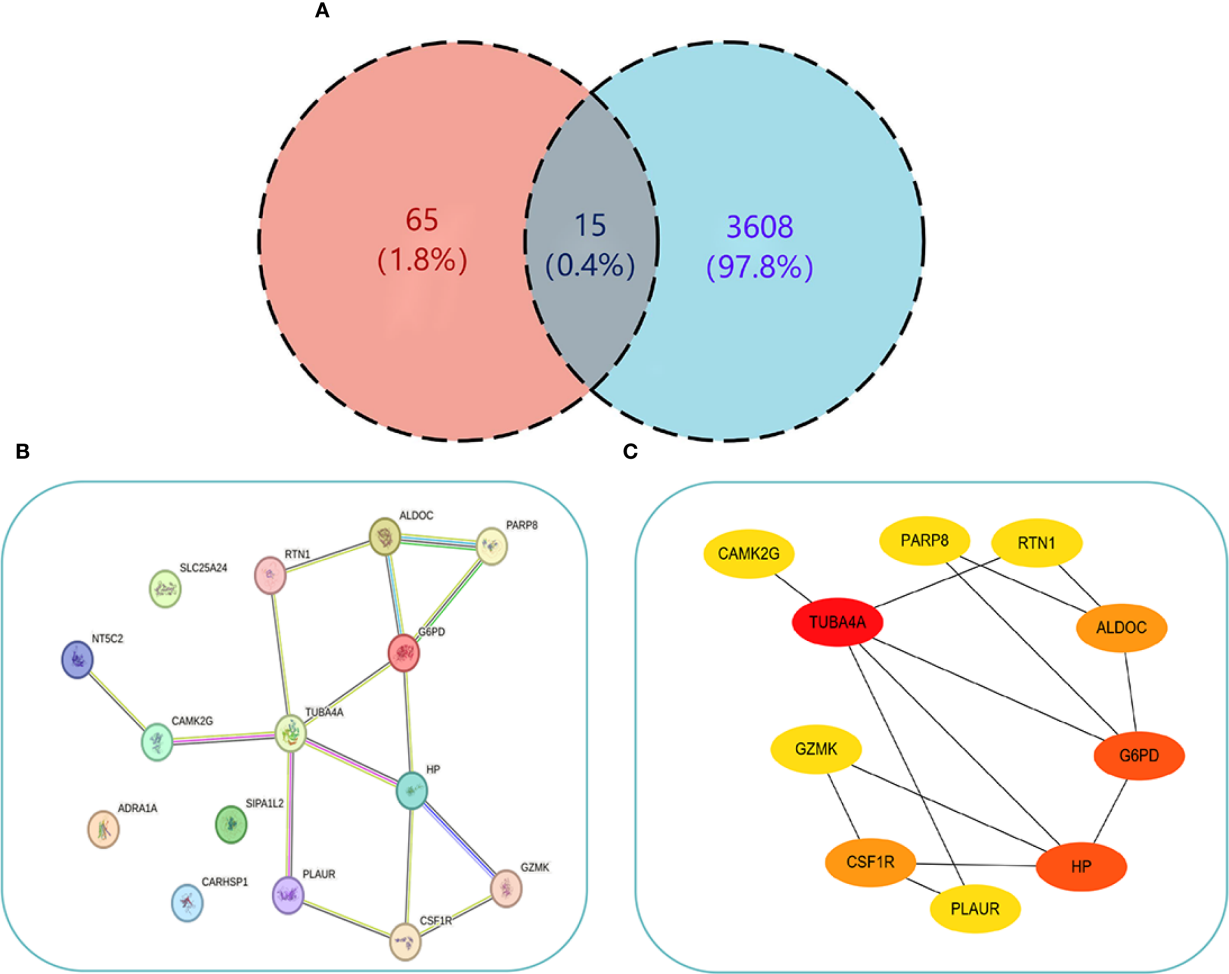
PPI Network interaction relationships. **(A)** Venn diagram showing the intersection of genes from both diseases and ARDEGs. **(B)** PPI interaction network of the 15 common genes obtained from STRING. **(C)** Visualization of the PPI network using Cytoscape (node color intensity indicates higher Degree values). .

To uncover the relation of cellular senescence to the pathogenesis of PCOS and T2DM, this study intersected the gene sets of both diseases with senescence-related genes, resulting in 15 common genes - ARDEGs (**Figure 5A**).

To explore the PPI relationships among the 15 proteins, the genes were uploaded to STRING to construct a PPI network (**Figure 5B**). The network included 11 nodes and 15 interaction pairs, which were subsequently visualized using Cytoscape (**Figure 5C**). Hub ARDEGs were pinpointed via the CytoHubba plugin, revealing 10 hub genes, including the downregulated gene GZMK, and the upregulated genes TUBA4A, G6PD, HP, CSF1R, ALDOC, RTN1, PARP8, PLAUR, and CAMK2G (**Table 2**). These hub genes are associated with immune cell function, glycolysis, and nucleotide phosphorylation and participate in various metabolic processes. Furthermore, CSF1R, TUBA4A, and PLAUR are implicated in apoptosis, migration, and adhesion.

**Table 2:** Gene information of the top 10 by degree.

NO.	Gene symbol	Full name	Function
1	TUBA4A	Tubulin Alpha 4A	TUBA4A may play a pivotal role in maintaining cell morphology, facilitating cell division, intracellular transport, and signal transduction.
2	G6PD	Glucose-6-Phosphate Dehydrogenase	G6PD catalyzes the conversion of glucose-6-phosphate to 6-phosphoglucono-δ-lactone, producing reduced nicotinamide adenine dinucleotide phosphate (NADPH) as a hydrogen donor, and is involved in various metabolic processes within the body.
3	HP	Haptoglobin	HP is widely present in the blood and body fluids of humans and many other mammals. It has the ability to bind free hemoglobin to form complexes and, as an acute-phase protein, participates in host defense against infection and tissue repair.
4	CSF1R	Colony Stimulating Factor 1 Receptor	CSF1R promotes the survival and activation of monocytes and macrophages.
5	ALDOC	Aldolase C	Aldose reductase plays a key role in carbohydrate metabolism by participating in the reduction of aldoses to their corresponding alcohols.
6	RTN1	Reticulon 1	RTN1 plays a critical role in membrane trafficking and secretion in neuroendocrine cells, and may serve as a potential diagnostic and therapeutic marker for malignant tumors with neuroendocrine components.
7	PARP8	Poly(ADP-ribose) Polymerase Family Member 8	PARP8 may regulate protein function, DNA repair, or signal transduction by catalyzing ADP-ribosylation modifications on target proteins.
8	PLAUR	Plasminogen Activator, Urokinase Receptor	PLAUR binds to urokinase-type plasminogen activator (uPA), promoting degradation of the extracellular matrix and cell migration, and plays a key role in tumor invasion and metastasis.
9	GZMK	Granzyme K	GZMK has the ability to recognize, bind, and lyse specific target cells, protecting the host by lysing cells that display non-self antigens on their surface.
10	CAMK2G	Calmodulin-Dependent Protein Kinase II Gamma	CAMK2G may regulate the expression of proinflammatory cytokines (such as IL-6 and TNF-α) via the NF-κB signaling pathway and is involved in the immunometabolic reprogramming of immune cells.

### 3.5 Validation of differential expression of hub genes and ROC curve analysis

**Figure 6:**
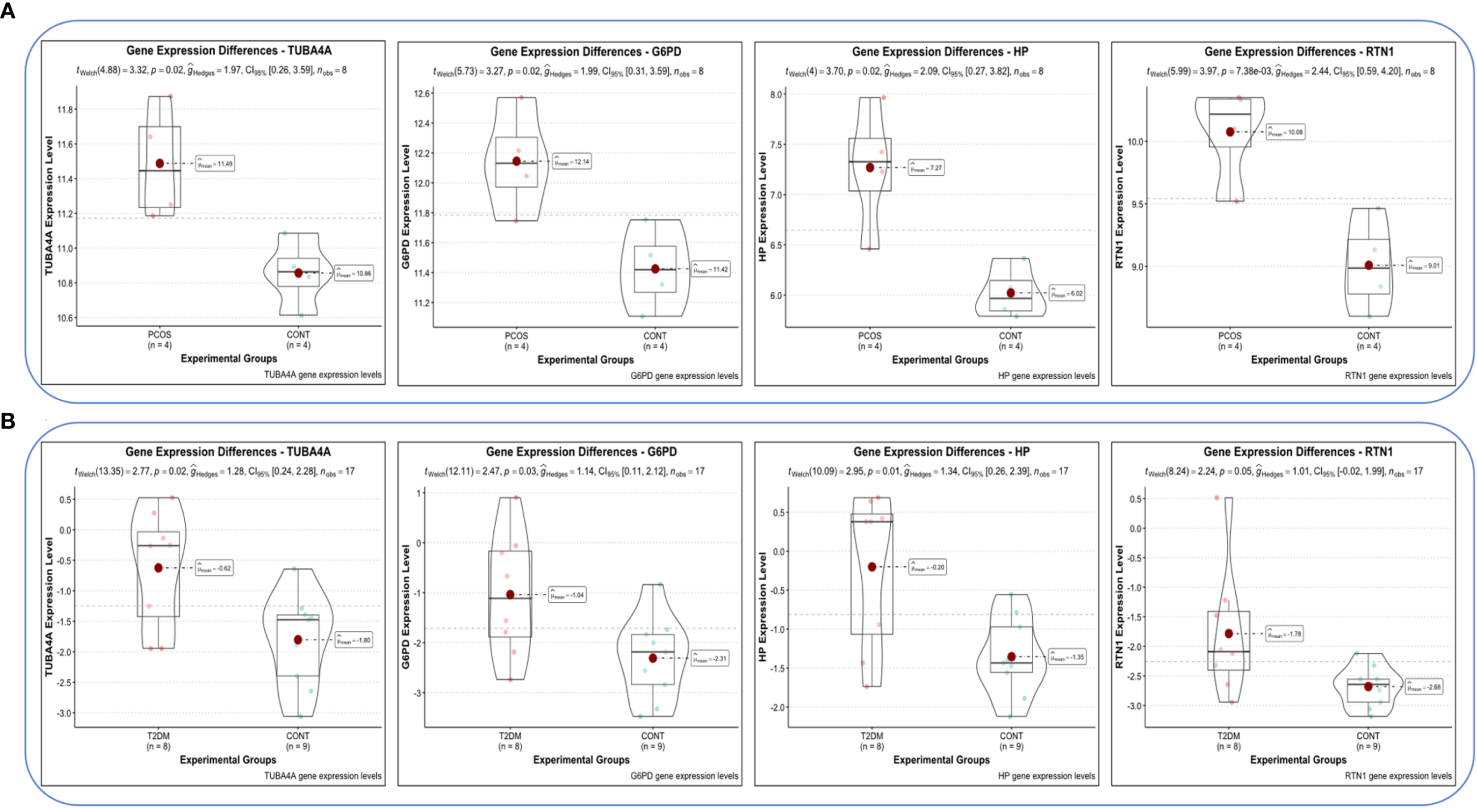
Expression levels of hub genes in both diseases. **(A)** Expression levels of hub genes in GSE54248. **(B)** Expression levels of hub genes in GSE23561.

**Figure 7:**
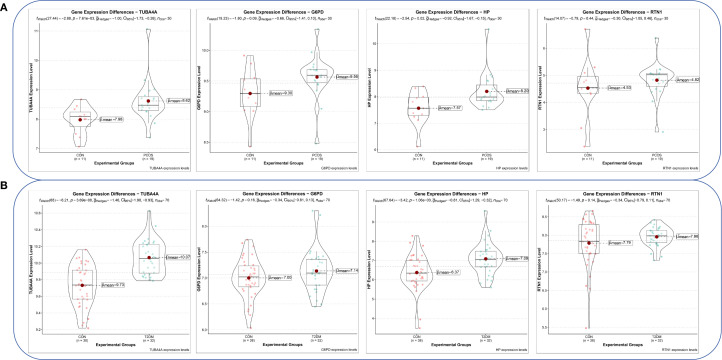
Expression levels of hub genes in the PCOS_GC and T2DM_PBMC datasets. **(A)** Expression levels of hub genes in the PCOS_GC dataset. **(B)** Expression levels of hub genes in the T2DM_PBMC dataset.

**Figure 8:**
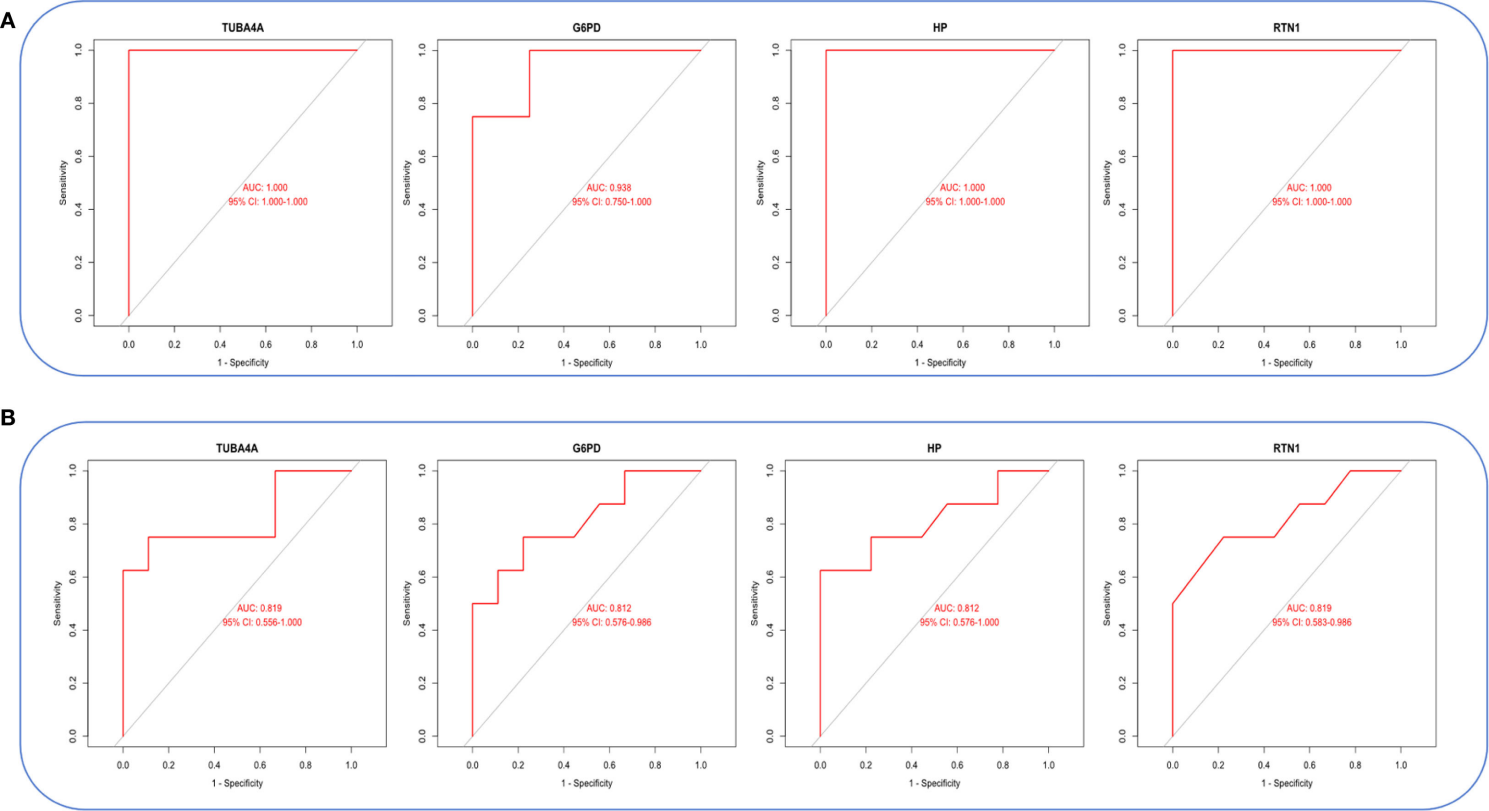
ROC curves of hub genes in both diseases. **(A)** ROC curves of hub genes in GSE54248. **(B)** ROC curves of hub genes in GSE23561.

**Figure 9:**
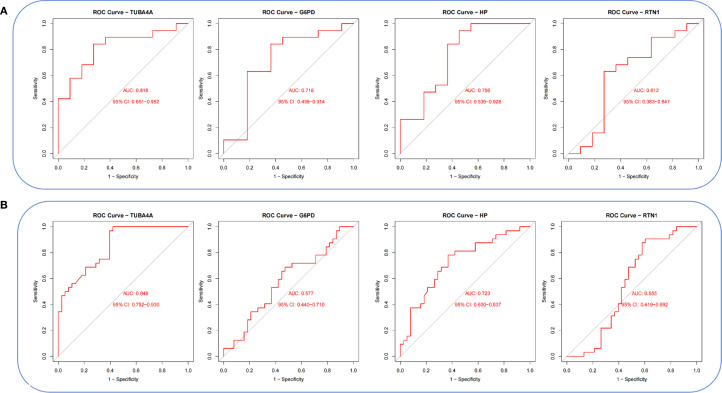
ROC curves of hub genes in the integrated PCOS_GC and T2DM_PBMC datasets. **(A)** ROC curves of hub genes in the PCOS_GC dataset. **(B)** ROC curves of hub genes in the T2DM_PBMC dataset.

Violin plots were constructed to visualize the expression levels of key genes in PCOS and T2DM (**Figures 6A, B**). The expression levels of hub genes were significantly upregulated in both PCOS and T2DM patients, which was largely consistent with the findings in the validation cohort (**Figures 7A, B**). Although the expression of RTN1 and G6PD did not reach statistical significance, the observed trends remained consistent with previous results. ROC curve analysis of the diagnostic markers demonstrated excellent diagnostic performance for TUBA4A (PCOS_AUC=1; T2DM_AUC=0.819), RTN1 (PCOS_AUC=1; T2DM_AUC=0.819), G6PD (PCOS_AUC=0.938; T2DM_AUC=0.812), and HP (PCOS_AUC=1; T2DM_AUC=0.812) in both PCOS and T2DM cohorts (95% CI: 0.80-1) (**Figures 8A, B**). Validation using the PCOS_GC_DATASET and T2DM_PBMC_DATASET confirmed that these hub genes generally exhibited strong diagnostic performance (**Figures 9A, B**). Importantly, the expression profiles of these genes will be further validated in an independent clinical cohort using qRT–PCR. Expression patterns of the remaining key genes are presented in **Supplementary Figures 1**, **2**.

### 3.6 Single-gene GSEA

**Figure 10:**
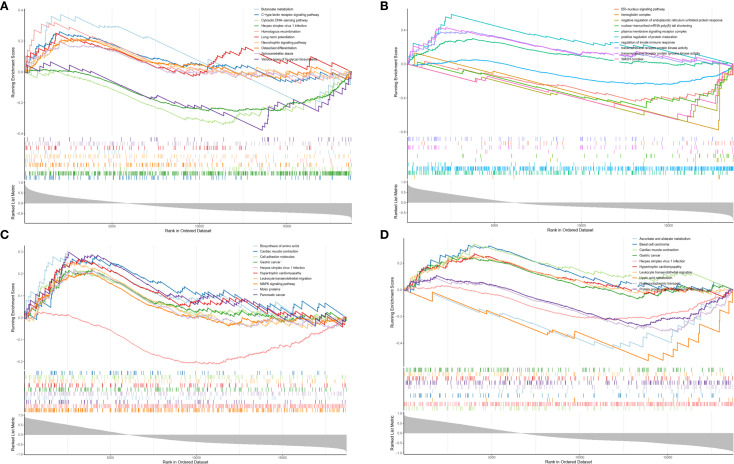
Enrichment analysis results of four key genes. **(A)** Enrichment analysis of RTN1. **(B)** Enrichment analysis of TUBA4A. **(C)** Enrichment analysis of HP. **(D)** Enrichment analysis of G6PD.

In this study, KEGG and GO analyses of TUBA4A, RTN1, HP, and G6PD revealed the following: RTN1 is notably enriched in the butyrate metabolism pathway, TUBA4A is markedly involved in the positive regulation of protein maturation, HP is significantly enriched in nucleotide transferase activity, and G6PD is significantly enriched in the endoplasmic reticulum(ER) regulation of Golgi vesicle-mediated transport pathway. Therefore, the foregoing four genes are critical in intracellular material synthesis, modification, transport, and functional regulation (**Figures 10A–D**).

### 3.7 Analysis of immune cell infiltration landscape in PCOS patients

**Figure 11:**
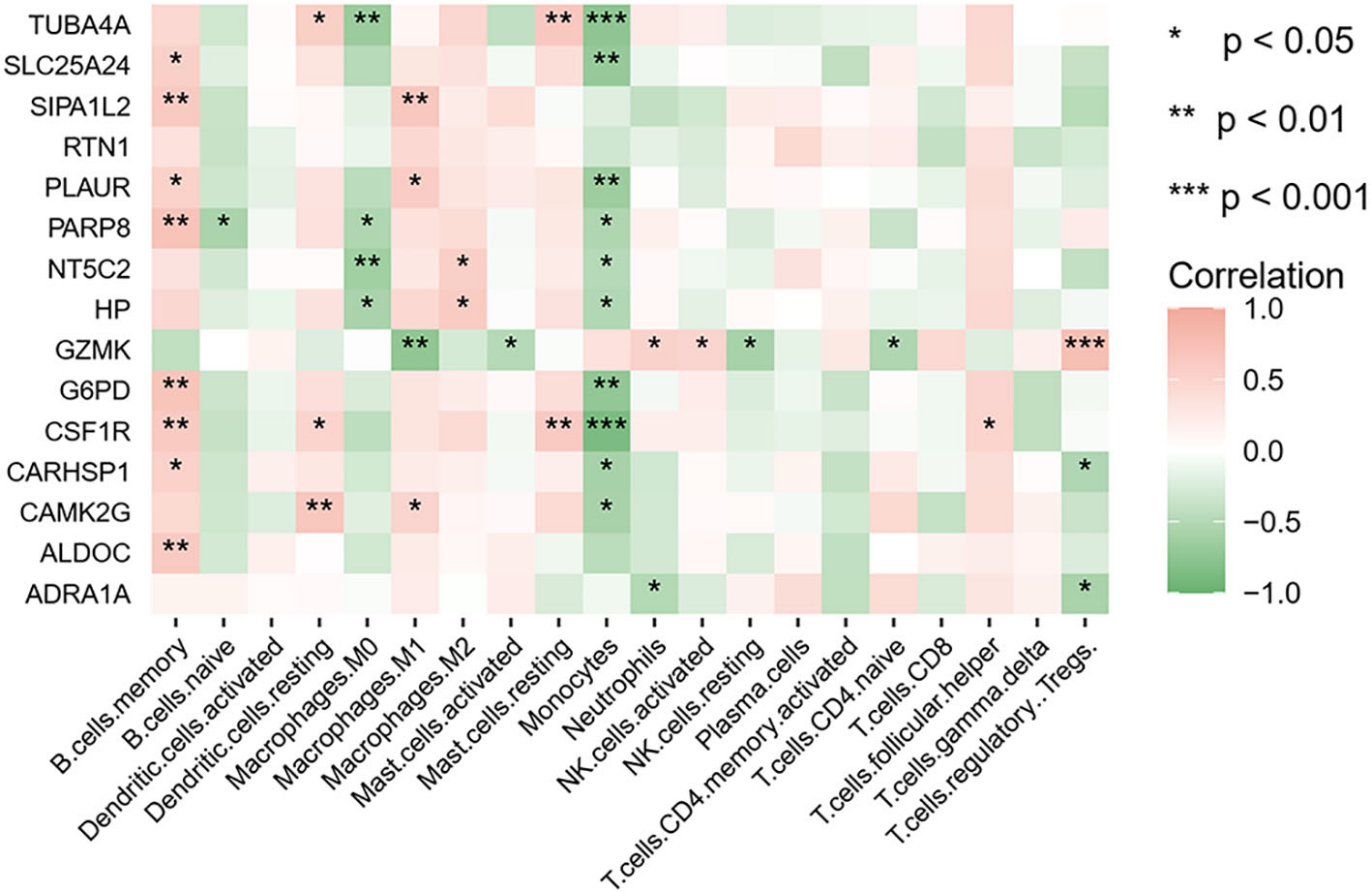
Heatmap of the correlation between key genes and immune cells. Red and green indicate positive and negative correlations, respectively. *p < 0.05; **p < 0.01; ***p < 0.001.

Given the pivotal role of immune activation in PCOS, our study investigated the interactions between immune cells and hub genes. The infiltration of 20 distinct immune cell types and their correlation with key genes was assessed using the CIBERSORT algorithm. Spearman correlation analysis revealed that in PCOS, TUBA4A and HP were negatively correlated with M0 macrophages and monocytes, whereas TUBA4A exhibited a positive relation to resting dendritic cells and resting mast cells. HP, on the other hand, showed a positive correlation with M2 macrophages. G6PD was significantly positively correlated with memory B cells and negatively correlated with monocytes (**Figure 11**). All p-values were < 0.05. These results further elucidate the relationship between immune cell infiltration and key genes in PCOS.

### 3.8 Single-cell analysis of hub gene locations

**Figure 12:**
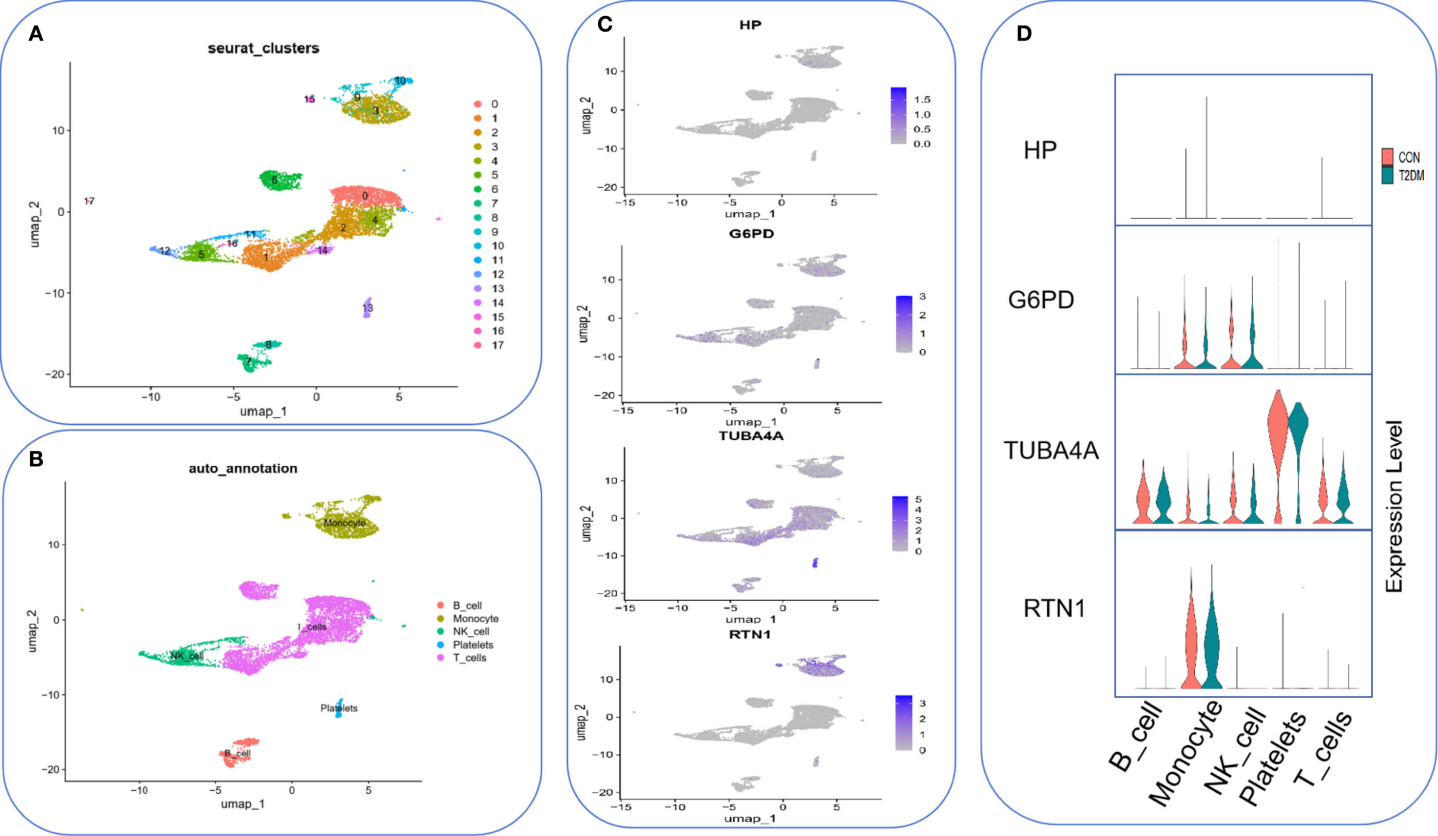
Single-cell expression profiling of core genes in T2DM peripheral blood. **(A)** 18 subpopulations of peripheral blood single-cell transcriptomes from T2DM (n=4). **(B)** Cell type annotation. **(C)** Expression hotspot distribution of core genes on UMAP plots. **(D)** Violin plots presenting key gene expressions across different cell types. Red indicates the control group, and green indicates the T2DM group.

Quality control was first performed on four samples from the T2DM dataset (GSE280401) and the Seurat standard pipeline to classify all cells into 18 subpopulations (**Figure 12A**). Cell type annotation was performed using SingleR (v1.6.1), and five major cell types were identified: monocytes, NK cells, platelets, B cells, and T cells (**Figure 12B**). Cell-type-specific expression analysis of the core genes demonstrated that G6PD is specifically expressed in monocytes and NK cells, TUBA4A exhibits a broad expression profile but is significantly elevated in platelets, and RTN1 is primarily expressed in monocytes (**Figures 12C, D**).

### 3.9 Quantitative real-time PCR experiments

**Figure 13:**
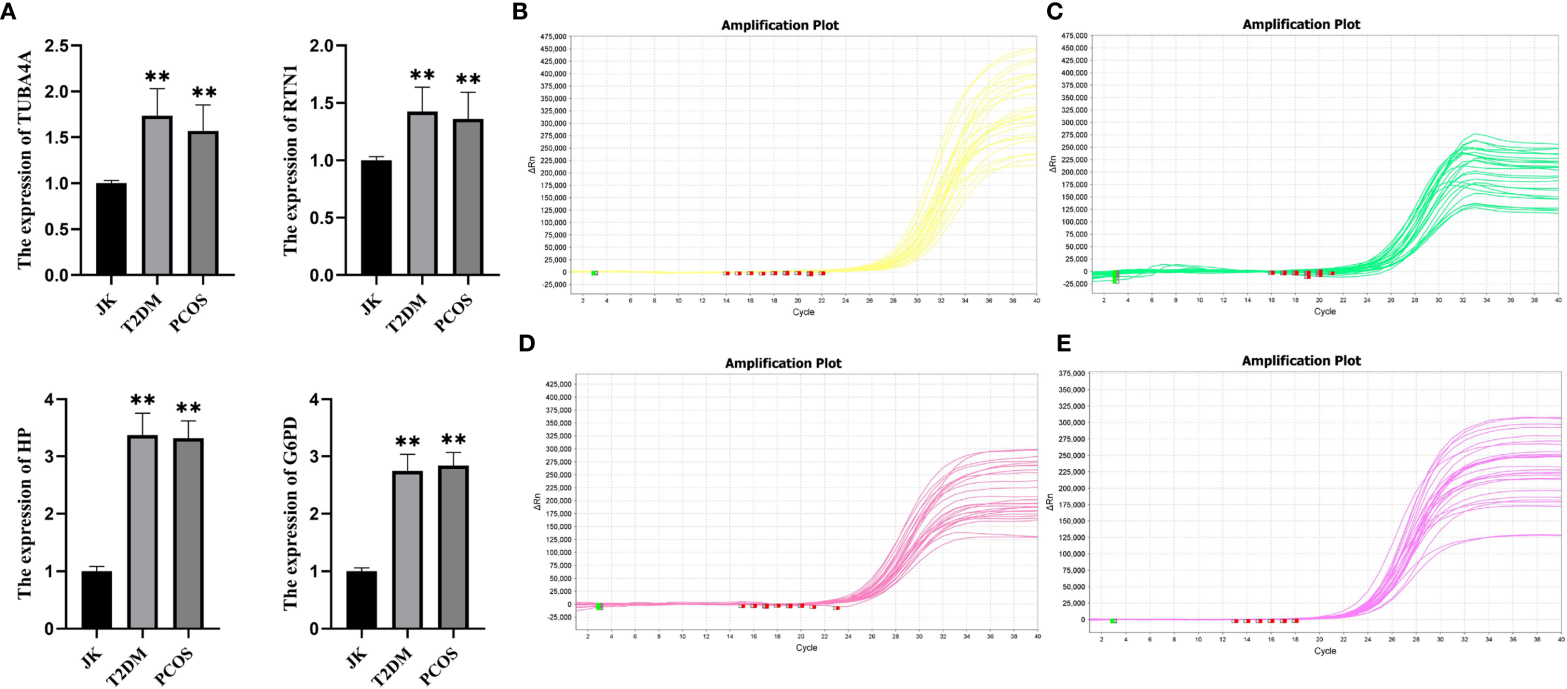
qRT–PCR validation results. **(A)** JK represents the healthy control group, T2DM represents the type 2 diabetes group, and PCOS represents the PCOS group. Bar graphs display the significantly higher expression levels of key genes in T2DM and PCOS patients compared to healthy controls. **(B)** Expression levels of G6PD across the three groups. **(C)** Expression levels of HP across the three groups. **(D)** Expression levels of RTN1 across the three groups. **(E)** Expression levels of TUBA4A across the three groups. *p < 0.05; **p < 0.01; ***p< 0.001, compared with the JK group.

qRT–PCR validation of nine peripheral blood samples corroborated the data analysis, with results indicating that HP, G6PD, TUBA4A, and RTN1 were all highly expressed in the peripheral blood of PCOS and T2DM sufferers (**Figures 13B–E**), with expression levels significantly higher than those in healthy controls (**Figure 13A**).

## 4 Discussion

PCOS, a common reproductive endocrine disorder in women of reproductive age, is characterized by IR and hyperinsulinemia, which represent a prediabetic state for T2DM (31). Endogenous IR in T2DM patients can stimulate ovarian granulosa cells, collectively promoting small follicle growth and increased follicle numbers, thereby contributing to the development of PCOS (32). Furthermore, the synergistic action of insulin and luteinizing hormone can elevate cellular androgen levels, resulting in significant clinical, biochemical, and metabolic similarities between T2DM and PCOS (11). In recent years, T2DM onset in women has trended toward younger ages, and prolonged disease duration during the reproductive period significantly exacerbates the risk of reproductive system damage (33). Accordingly, the comorbid mechanisms of these two diseases have been a major focus of research. Some studies have suggested that elevated glucose-dependent insulinotropic peptide (GIP) and reduced glucagon-like peptide-1 (GLP-1) may serve as early biomarkers for the progression of PCOS to T2DM (34). Other studies indicate that decreased levels of metabolic dysregulation markers, such as sex hormone-binding globulin (SHBG), represent a key risk factor for T2DM in women with PCOS (35). These findings underscore the complexity of shared pathophysiological mechanisms, highlighting the need to identify common pathological hubs to enable precise diagnosis and targeted interventions.

Initially, 80 overlapping DEGs were identified between PCOS and T2DM datasets, including 76 commonly upregulated genes and 4 commonly downregulated genes. Functional annotation of the upregulated genes revealed enrichment in cellular signaling and communication, metabolism and energy homeostasis, cell structure and motility, protein modification and degradation, immunity and inflammation, transcriptional and epigenetic regulation, as well as cell growth, differentiation, and development. Downregulated genes were primarily associated with immune responses, protein degradation and processing, promotion of inflammatory responses, and involvement in sulfation modification processes. GO and KEGG pathway enrichment analyses indicated that these DEGs were significantly enriched in immune responses mediated by specific granulocytes and calcium signaling pathways. Moreover, emerging evidence suggests a close relationship between immune responses and cellular senescence: endogenous senescence-inducing factors may participate in terminal differentiation of immune cells (36), while regulatory T (Treg) cells can suppress immune responses through senescence induction (10). Simultaneously, calcium homeostasis imbalance, such as mitochondrial Ca^2+^ overload caused by enhanced ER-mitochondria coupling, is considered a critical driver of cellular senescence (37).

Cellular senescence is an irreversible state of cell cycle arrest triggered by stress, accompanied by loss of proliferative and differentiation capacity and progressive decline in physiological function, ultimately compromising tissue homeostasis (38). Hallmarks of senescence include elevated senescence-associated β-galactosidase (SA-β-gal) activity, upregulation of cell cycle inhibitors (p16/p21/p27) (39), and the SASP, characterized by the release of chemokines (e.g., CCL2, IL-8, IL-6), growth factors (e.g., TGF-β), and matrix-remodeling enzymes (e.g., MMPs) (40, 41). Studies indicate that metabolic aging is accelerated in PCOS patients, manifested by premature worsening of IR and β-cell dysfunction, which closely overlap with physiological aging processes (42). A local hyperandrogenic ovarian microenvironment can directly induce granulosa cell (GC) senescence or indirectly mediate GC senescence through activation of ER stress (ER stress). Concurrently, systemic effects of cellular senescence, including obesity and inflammation, promote the onset and progression of T2DM (43). This establishes a vicious cycle: the diabetic microenvironment facilitates the generation and accumulation of senescent cells, whose metabolic dysfunction both increases senescence formation and impairs their clearance; conversely, senescent cells contribute to T2DM-associated tissue dysfunction and comorbidities (19).

In light of the foregoing, the co-expressed genes of both diseases with senescence factors were further intersected, resulting in the identification of 15 pivotal genes. To further elucidate the most diagnostically valuable key genes, these 15 genes were uploaded to the STRING database to construct a PPI network. Hub ARDEGs were identified via the CytoHubba plugin in Cytoscape, ultimately revealing 10 hub ARDEGs. Among them, TUBA4A (a microtubule stability regulator), RTN1 (an ER stress regulatory factor), G6PD (a redox homeostasis hub), and HP (a hemoglobin-scavenging and antioxidant protein) exhibited robust diagnostic performance, which was confirmed via ROC curve analysis. Moreover, real-time quantitative PCR experiments demonstrated that these four genes in the peripheral blood of PCOS and T2DM sufferers were markedly upregulated in contrast to those in healthy individuals. These findings suggest that these four genes have clear diagnostic value for both PCOS and T2DM, providing molecular evidence for the clinical diagnosis of these conditions and indicating a close relationship between senescence-related pathological mechanisms and the pathogenesis of PCOS and T2DM.

Tubulin Alpha 4A (TUBA4A), a key gene encoding α-tubulin, participates in diverse intracellular processes by regulating microtubule dynamics. TUBA4A is broadly expressed across human tissues, with particularly high expression in the brain. Smith et al., through whole-exome sequencing and related experiments, identified TUBA4A as a potential pathogenic gene for familial amyotrophic lateral sclerosis (FALS), highlighting its diagnostic significance for FALS (44). Li et al. confirmed that TUBA4A is essential for spindle assembly during oocyte meiosis and zygotic mitosis, and that pathogenic mutations can lead to zygotic arrest and early embryonic developmental failure, representing a novel genetic risk factor for infertility (45). The present study reports for the first time that TUBA4A is significantly upregulated in PCOS patients, suggesting that it may be one of the effective genes contributing to infertility in PCOS. Considering the increased risks of miscarriage, preterm birth, and other adverse pregnancy outcomes in PCOS patients (46), TUBA4A may participate in pathological processes leading to poor pregnancy outcomes through interference with embryonic development, indicating its potential as a diagnostic biomarker. Furthermore, Smith et al. demonstrated that TUBA4A expression increases markedly in an age-dependent manner (>50-fold) (44) and that its pathogenic mutations may accelerate aging-related pathological processes, precipitating the onset of late-onset diseases (47). Collectively, our study further validates, through cellular senescence phenotype analysis, that TUBA4A may serve as a key molecular driver of aging, providing experimental evidence for elucidating the aging mechanisms underlying PCOS/T2DM comorbidity.

Reticulon 1 (RTN1), an ER-resident protein-coding gene, modulates ER stress and is involved in various pathological processes, serving as a diagnostic biomarker for neuroendocrine disorders and cancer (48–50). In chronic kidney disease models, such as unilateral ureteral obstruction and diabetic nephropathy, elevated RTN1 induces ER stress, promoting fibrosis and tissue damage (51). Importantly, in the reproductive-metabolic field, RTN1 has been identified as a potential immunotherapeutic target for endometrial cancer and PCOS (52). Our study further confirms its significant upregulation in PCOS patients. Interestingly, another study reported contrasting findings, in which RTN1 deficiency in mice did not result in overt fertility defects (50). Additionally, RTN1 is an ER stress-associated DEG in T2DM, participating in protein folding and metabolic regulation, with predictive value for disease occurrence (53). Mechanistic studies indicate that RTN1 regulates apoptosis via ER stress mediation (51), while persistent ER stress can induce senescence phenotypes (54, 55). Considering our findings that RTN1 is significantly upregulated in both T2DM and senescent cell models, it is hypothesized that RTN1 may serve not only as a potential diagnostic biomarker for PCOS/T2DM comorbidity but also as a direct driver of disease progression through the ER stress-senescence axis.

Glucose-6-phosphate dehydrogenase (G6PD), the rate-limiting enzyme of the pentose phosphate pathway (PPP), regulates redox homeostasis via Nicotinamide Adenine Dinucleotide Phosphate Hydrogen (NADPH) (56). Its functional complexity is reflected in multiple aspects: (i) through PPP activation, G6PD may promote tumorigenesis (e.g., hepatocellular carcinoma, glioma, and breast cancer) and non-neoplastic diseases (e.g., malaria and life-threatening hemolytic anemia) (57, 58); (ii) through brain peroxide metabolism, G6PD participates in Alzheimer’s disease pathogenesis (59). Notably, reduced G6PD activity in a mouse model of endometriosis enhances copper toxicity (60). Moreover, G6PD-dependent NADPH generation regulates the activity of multiple redox-sensitive factors, including Nrf2, NF-κB, HIF-1α, and AMPK (61), which are directly implicated in oxidative stress, inflammation, and hyperandrogenemia in PCOS. Some studies have suggested that G6PD deficiency may constitute a risk factor for diabetes (62, 63), likely due to increased protein oxidation and lipid peroxidation (64). Limited literature has also linked G6PD deficiency to cellular senescence, indicating that insufficient G6PD activity predisposes cells to growth retardation and death (65). Our study further confirms that G6PD is upregulated in PCOS/T2DM and senescent cells, consistent with prior research. Based on these findings, G6PD may contribute to the pathogenesis of PCOS/T2DM and cellular aging by modulating redox homeostasis, providing novel evidence for its potential molecular diagnostic value in reproductive-metabolic comorbidity.

Haptoglobin (HP) is the principal plasma scavenger of hemoglobin (Hb), regulating the clearance of circulating Hb via the macrophage-specific receptor CD163 and thereby preventing Hb-mediated deleterious effects (66). The core antioxidative mechanism of HP lies in its binding to free Hb to form the HP-Hb complex. This complex not only mitigates lipid peroxidation-induced endothelial damage by inhibiting the oxidative modification of low-density lipoprotein (LDL) but is also rapidly cleared by the mononuclear phagocyte system through CD163-mediated endocytosis. Such clearance prevents glomerular filtration of Hb (thereby protecting against tubular injury) and suppresses Hb-mediated tissue oxidative toxicity (67). Moreover, HP can attenuate hyperlipidemia-induced oxidative stress by neutralizing the oxidative activity of extracellular Hb, thereby delaying the progression of T2DM and its complications (68, 69). In diabetic models, dynamic changes in HP expression closely correlate with serum levels of TNF-α and IL-6, as well as the TNF-α/IL-6 ratio (8-week follow-up data) (70). Proteomic analyses have further demonstrated that total HP and the HP-β chain are significantly elevated in the serum of patients with PCOS and positively correlate with C-reactive protein levels (71), suggesting that HP, as an acute-phase protein, plays a central role in the inflammatory processes of PCOS (72, 73). Taken together with our findings of marked HP upregulation in PCOS and T2DM, HP may contribute to the pathogenesis of these conditions via modulation of inflammatory responses and oxidative stress.

To further elucidate the relationship between key genes, disease states, and aging-related factors, functional enrichment analyses were performed on HP, G6PD, TUBA4A, and RTN1. The results indicated that RTN1 is significantly enriched in the butyrate metabolism pathway. Butyrate, a short-chain fatty acid, activates gut epithelial GPCR41 receptors, promoting the secretion of gut hormones such as glucagon-like peptide and serotonin (74), thereby exerting anti-inflammatory effects, enhancing insulin sensitivity, and maintaining intestinal barrier integrity (75). Experimental evidence shows that oral sodium butyrate markedly improves insulin sensitivity in diabetic mice, and the butyrate content in PCOS patients is significantly lower than in controls (76). These observations suggest that RTN1 may play a central role in PCOS/T2DM pathogenesis by regulating butyrate metabolism, influencing insulin secretion, and modulating gut microbiota function, consistent with the experimental findings of the present study. Functional enrichment analysis also revealed that G6PD is significantly enriched in the ER-Golgi vesicle transport pathway. Given that ER stress has been confirmed to induce metabolic dysfunction and IR (77, 78), and considering the previously described central role of ER stress in PCOS/T2DM comorbidity and cellular senescence (e.g., via RTN1 mechanisms), G6PD may contribute to the progression of cellular senescence underlying PCOS-T2DM comorbidity through regulation of ER/Golgi redox homeostasis. Moreover, HP was significantly upregulated in nucleotide transferase activity. Given the previously noted antioxidant and oxidative stress-inhibiting properties of HP, it is hypothesized that HP possibly delays disease progression and cellular senescence.

Considering the crucial role of immune cells in PCOS, the immune cell infiltration patterns in both diseases were analyzed via the CIBERSORT algorithm, and further evaluated the correlation between immune cells and key genes via Spearman correlation analysis. Macrophages, central to innate immunity, play an essential role in systemic inflammation and are distributed across all stages of the ovarian follicle (79). Our findings suggest that in PCOS, TUBA4A and HP are negatively correlated with M0 macrophages, indicating an imbalance in the immune regulatory mechanisms in PCOS patients, which promotes the onset of inflammatory responses and exacerbates the pathological processes of PCOS. Additionally, TUBA4A is positively correlated with resting dendritic cells and resting mast cells, suggesting that elevated expression of TUBA4A may facilitate the maintenance of these immune cells in their resting state, inhibiting their activation and interfering with the immune system, ultimately impairing ovarian function and influencing the development of PCOS. M2 macrophages play a role in the initiation and progression of IR (80). In this study, HP was found to be positively correlated with M2 macrophages (Macrophages.M2), leading us to hypothesize that metabolic abnormalities and inflammatory responses in PCOS may be closely linked to the upregulation of M2 macrophages. These findings reflect the intricate relationship between immune dysregulation and metabolism in PCOS, offering new perspectives and approaches for the diagnosis, treatment, and prognosis of the disease.

Finally, single-cell datasets from T2DM patients were downloaded, and single-cell annotation analysis was performed to investigate cellular heterogeneity and elucidate potential underlying mechanisms. The results revealed that TUBA4 exhibited significant enrichment in platelets, RTN1 was highly expressed specifically in the monocyte population, and G6PD demonstrated dual-positive expression in both monocytes and natural killer (NK) cells. These findings highlight the cell-specific dynamic regulatory networks of key genes within the T2DM immune microenvironment. This suggests that these genes may contribute to the pathological progression of T2DM by modulating platelet activation (TUBA4), inflammatory cascades (RTN1), and redox homeostasis (G6PD). These insights provide molecular evidence for T2DM pathogenesis and offer new biological markers and directions for translational research in precision medicine.

In summary, this study identified shared co-expressed genes between PCOS and T2DM, analyzed potential pathogenic mechanisms, and integrated these diseases with cellular senescence. Four key genes, TUBA4A, RTN1, HP, and G6PD, CAN serve as potential biomarkers. Additionally, real-time qRT–PCR experiments were conducted on these key genes, and the results were consistent with the data analysis, confirming their diagnostic value for both PCOS and T2DM. This study has certain limitations. The causal relationship between hub genes and cellular senescence requires further validation through functional experiments. In future studies, it is our plan to knock down these key genes in cell lines and assess their effects on cellular senescence markers (e.g., p16, SA-β-gal) and SASP secretion. Additionally, due to tissue-specific differences, the peripheral blood immune profile may not fully reflect the ovarian microenvironment. Therefore, our findings will be validated in ovarian tissue samples to enhance the reliability of our conclusions.

## 5 Conclusion

Through the discovery of four shared senescence-associated genes, cellular senescence was established as a potential mechanistic link between PCOS and T2DM. These findings unveil previously unrecognized molecular connections between the two diseases and were experimentally validated, ensuring the accuracy of our results. This study provides novel targets and directions for the development of integrated diagnostic methods and therapeutic strategies based on cellular senescence mechanisms for both PCOS and T2DM.

## Data Availability

The raw data supporting the conclusions of this article will be made available by the authors, without undue reservation.

## Supplementary material

Supplementary Table 1 A list of 80 shared genes regulated by PCOS and T2DM..

Supplementary Figure 1 Expression levels and ROC curves of six hub genes in two diseases. **(A–F)** Expression levels and ROC curves of key genes in GSE54248. **(G–L)** Expression levels and ROC curves of key genes in GSE23561.

Supplementary Figure 2 Expression levels and AOC curves of hub genes in PCOS_GC_DATASET and T2DM_PBMC_DATASET. **(A–F)** Expression levels and AOC curves of hub genes in PCOS_GC_DATASET. **(G–L)** Expression levels and AOC curves of hub genes in T2DM_PBMC_DATASET.
