# Supplementary material for: Shared senescence-associated gene networks in PCOS and T2DM: biomarker identification and functional validation
Source: Front Endocrinol (Lausanne). 2025 Sep 25;16:1652178. doi: 10.3389/fendo.2025.1652178 (PMC12507634; doi:10.3389/fendo.2025.1652178)
Supplement: Supplementary Table 1 — A list of 80 shared genes regulated by PCOS and T2DM.. [file Table1.docx]

**Supplementary Materials**

**Supplementary Table 1** A list of 80 shared genes regulated by PCOS and T2DM

| Gene Name | PCOSvsCONT logFC | PCOSvsCONT P.Value | T2DMvsCONT logFC | T2DMvsCONT P.Value |
| --- | --- | --- | --- | --- |
| MCEMP1 | 1.86 | 0.0002 | 0.63 | 0.0488 |
| CR1 | 1.82 | 0.0040 | 1.05 | 0.0011 |
| SLC25A24 | 1.76 | 0.0071 | 1.24 | 0.0016 |
| CYP4B1 | 1.64 | 0.0148 | 0.91 | 0.0089 |
| PGM5 | 1.48 | 0.0245 | 0.87 | 0.0076 |
| PLAUR | 1.42 | 0.0023 | 0.75 | 0.0305 |
| BMX | 1.39 | 0.0176 | 0.66 | 0.0290 |
| BRWD3 | 1.31 | 0.0131 | 0.69 | 0.0179 |
| ECHDC3 | 1.29 | 0.0030 | 0.73 | 0.0130 |
| MAFF | 1.28 | 0.0146 | 0.82 | 0.0133 |
| SIPA1L2 | 1.27 | 0.0026 | 0.80 | 0.0203 |
| HP | 1.25 | 0.0027 | 1.03 | 0.0183 |
| PARP8 | 1.21 | 0.0005 | 1.05 | 0.0323 |
| NQO2 | 1.19 | 0.0098 | 0.84 | 0.0446 |
| GMPR2 | 1.18 | 0.0093 | 0.73 | 0.0198 |
| DOCK5 | 1.16 | 0.0013 | 0.92 | 0.0190 |
| TMLHE | 1.14 | 0.0004 | 1.17 | 0.0089 |
| SAMD8 | 1.12 | 0.0003 | 0.66 | 0.0224 |
| SLC22A4 | 1.09 | 0.0429 | 1.02 | 0.0054 |
| LSMEM1 | 1.08 | 0.0280 | 1.01 | 0.0242 |
| RTN1 | 1.07 | 0.0023 | 0.90 | 0.0183 |
| LRG1 | 1.05 | 0.0114 | 1.01 | 0.0042 |
| TUFT1 | 1.04 | 0.0128 | 0.67 | 0.0146 |
| NT5C2 | 1.01 | 0.0198 | 1.00 | 0.0018 |
| TMCO3 | 1.00 | 0.0055 | 1.47 | 0.0061 |
| RBMS2 | 0.98 | 0.0002 | 0.97 | 0.0036 |
| SLC38A2 | 0.97 | 0.0203 | 0.68 | 0.0257 |
| NLRX1 | 0.94 | 0.0038 | 0.61 | 0.0498 |
| MIR22HG | 0.94 | 0.0196 | 0.68 | 0.0188 |
| EXTL3 | 0.91 | 0.0052 | 1.11 | 0.0046 |
| CARHSP1 | 0.89 | 0.0013 | 0.98 | 0.0017 |
| FBXL2 | 0.89 | 0.0051 | 1.19 | 0.0003 |
| AGFG1 | 0.88 | 0.0089 | 1.01 | 0.0432 |
| CAMK2G | 0.88 | 0.0141 | 0.61 | 0.0351 |
| DNTTIP1 | 0.87 | 0.0047 | 1.72 | 0.0014 |
| ARAP3 | 0.86 | 0.0118 | 0.66 | 0.0042 |
| PKP4 | 0.86 | 0.0381 | 0.74 | 0.0330 |
| B3GNT8 | 0.85 | 0.0257 | 1.01 | 0.0074 |
| FAM53C | 0.85 | 0.0024 | 0.79 | 0.0235 |
| LATS2 | 0.84 | 0.0230 | 1.37 | 0.0042 |
| KATNIP | 0.84 | 0.0159 | 0.63 | 0.0401 |
| NCF4 | 0.84 | 0.0151 | 1.00 | 0.0226 |
| DNAJB12 | 0.83 | 0.0090 | 0.91 | 0.0083 |
| FXYD6 | 0.82 | 0.0030 | 1.16 | 0.0030 |
| ANKRD33 | 0.82 | 0.0168 | 1.11 | 0.0059 |
| CAMK1D | 0.80 | 0.0098 | 1.51 | 0.0009 |
| ANKRD11 | 0.80 | 0.0430 | 0.60 | 0.0399 |
| C5AR2 | 0.79 | 0.0425 | 1.24 | 0.0143 |
| ACOT9 | 0.79 | 0.0077 | 0.83 | 0.0130 |
| UBTD1 | 0.79 | 0.0299 | 0.65 | 0.0121 |
| IQSEC1 | 0.78 | 0.0133 | 1.42 | 0.0034 |
| CACNA1E | 0.76 | 0.0097 | 1.42 | 0.0007 |
| VDR | 0.75 | 0.0380 | 1.34 | 0.0008 |
| PTPRJ | 0.75 | 0.0104 | 0.68 | 0.0486 |
| CHRNA10 | 0.74 | 0.0383 | 0.99 | 0.0034 |
| SRA1 | 0.74 | 0.0044 | 0.95 | 0.0381 |
| G6PD | 0.72 | 0.0091 | 1.27 | 0.0086 |
| PRPF6 | 0.72 | 0.0033 | 0.71 | 0.0157 |
| SLC2A11 | 0.72 | 0.0319 | 1.26 | 0.0052 |
| KDM2A | 0.71 | 0.0490 | 1.10 | 0.0315 |
| IDS | 0.71 | 0.0107 | 0.75 | 0.0089 |
| ZNF503 | 0.70 | 0.0062 | 1.51 | 0.0054 |
| CARS2 | 0.69 | 0.0339 | 0.71 | 0.0339 |
| QSOX1 | 0.68 | 0.0111 | 1.25 | 0.0027 |
| RAB35 | 0.67 | 0.0023 | 0.61 | 0.0223 |
| ALDOA | 0.67 | 0.0224 | 0.78 | 0.0453 |
| CDH26 | 0.65 | 0.0294 | 1.05 | 0.0035 |
| ADRA1A | 0.65 | 0.0029 | 0.66 | 0.0383 |
| CSF1R | 0.65 | 0.0262 | 0.96 | 0.0085 |
| CATSPER1 | 0.65 | 0.0300 | 0.69 | 0.0458 |
| GHRL | 0.64 | 0.0249 | 1.20 | 0.0181 |
| ZFYVE1 | 0.64 | 0.0254 | 0.90 | 0.0110 |
| TUBA4A | 0.63 | 0.0108 | 1.05 | 0.0212 |
| TMEM25 | 0.62 | 0.0089 | 0.81 | 0.0302 |
| ALDOC | 0.59 | 0.0078 | 0.69 | 0.0397 |
| UPP1 | 0.59 | 0.0132 | 0.99 | 0.0036 |
| ORMDL3 | -0.64 | 0.0107 | -0.66 | 0.0254 |
| TYSND1 | -0.68 | 0.0137 | -0.74 | 0.0160 |
| GZMK | -1.02 | 0.0264 | -0.82 | 0.0128 |
| CHST12 | -0.97 | 0.0040 | -0.61 | 0.0433 |
