# Supplementary material for: Shared senescence-associated gene networks in PCOS and T2DM: biomarker identification and functional validation
Source: Front Endocrinol (Lausanne). 2025 Sep 25;16:1652178. doi: 10.3389/fendo.2025.1652178 (PMC12507634; doi:10.3389/fendo.2025.1652178)
Supplement: Supplementary Figure 1 — Expression levels and ROC curves of six hub genes in two diseases. (A–F) Expression levels and ROC curves of key genes in GSE54248. (G–L) Expression levels and ROC curves of key genes in GSE23561. [file Image1.pdf]

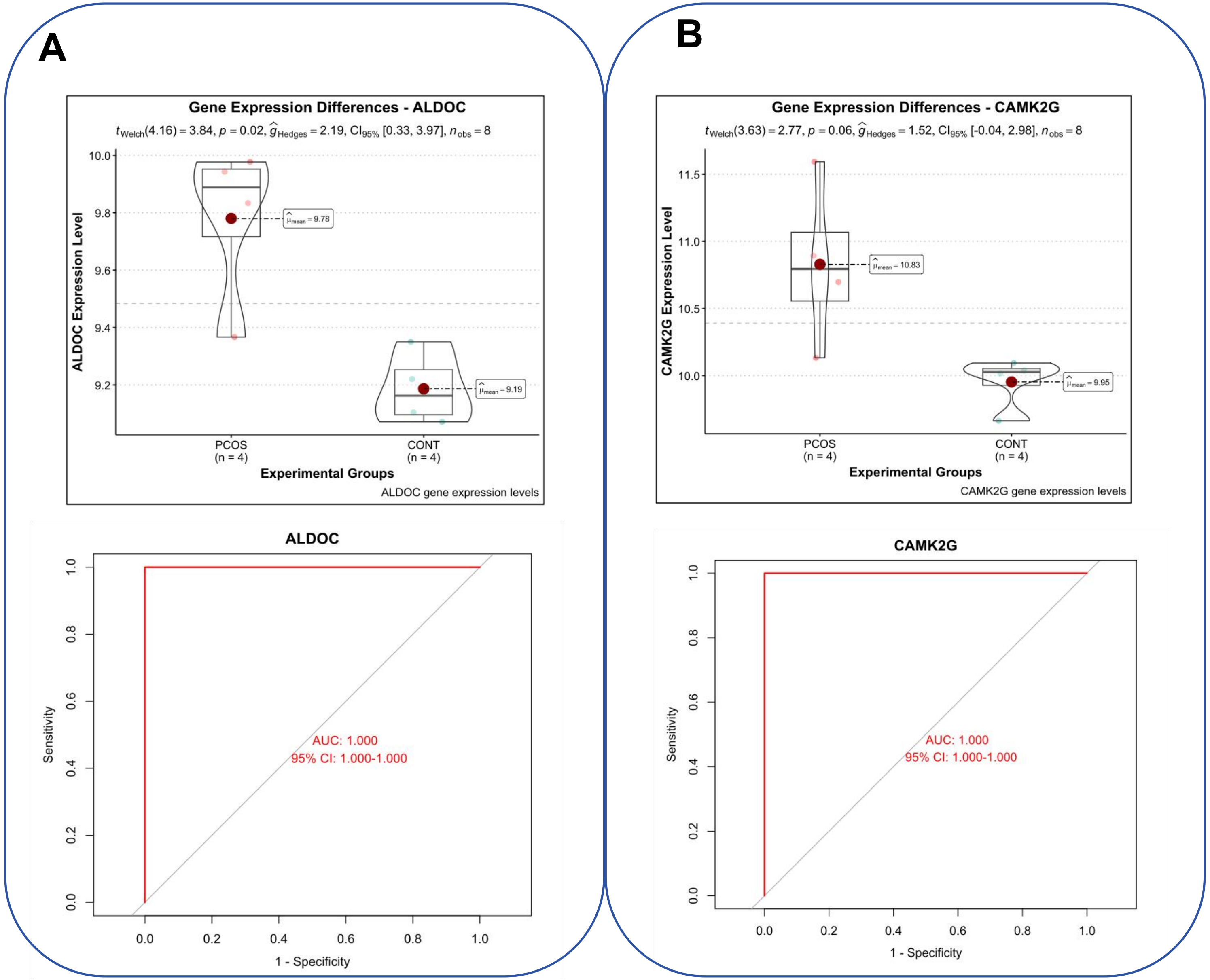

Supplementary Figure 1: Expression levels and ROC curves of six hub genes in two diseases
(A-F) Expression levels and ROC curves of key genes in GSE54248.
(G-L) Expression levels and ROC curves of key genes in GSE23561.

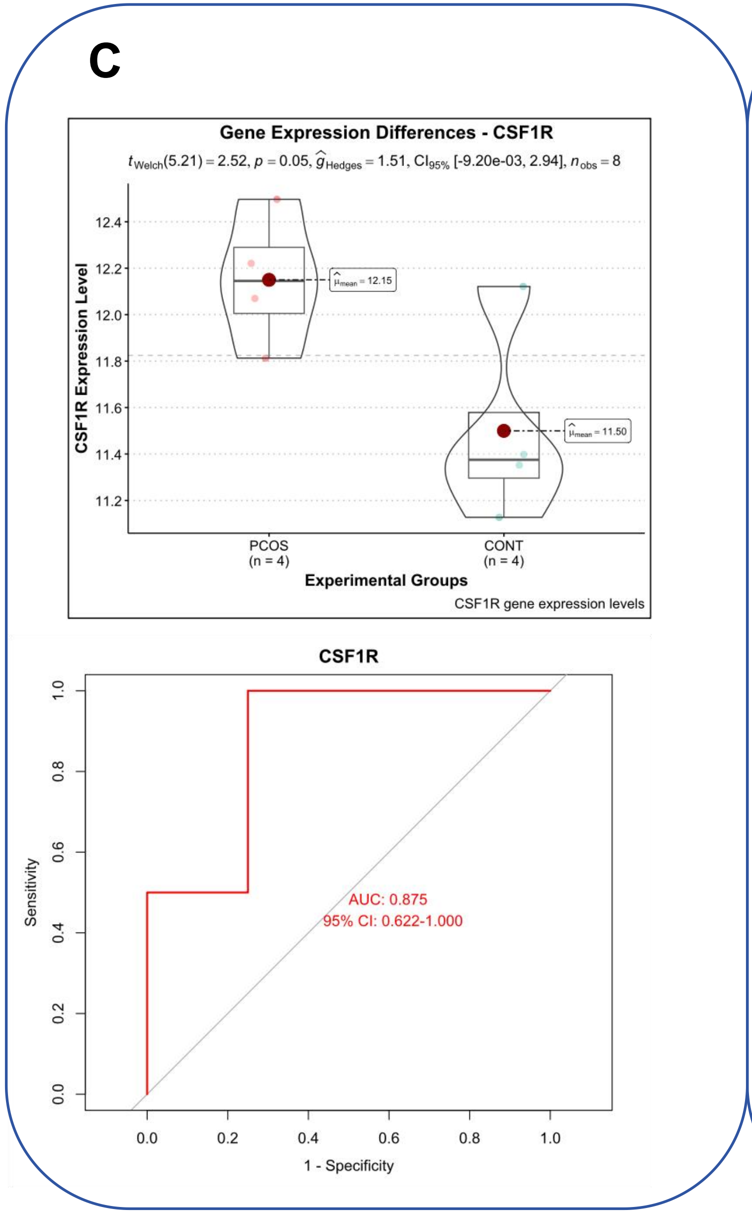
C
Gene Expression Differences - CSF1R
$t_{Welch}(5.21) = 2.52$, $p = 0.05$, $\hat{g}_{Hedges} = 1.51$, $CI_{95\%}$ [-9.20e-03, 2.94], $n_{obs} = 8$
CSF1R Expression Level
$\hat{\mu}_{mean} = 12.15$
$\hat{\mu}_{mean} = 11.50$
PCOS (n = 4)
CONT (n = 4)
Experimental Groups
CSF1R gene expression levels
CSF1R
Sensitivity
1 - Specificity
AUC: 0.875
95% CI: 0.622-1.000

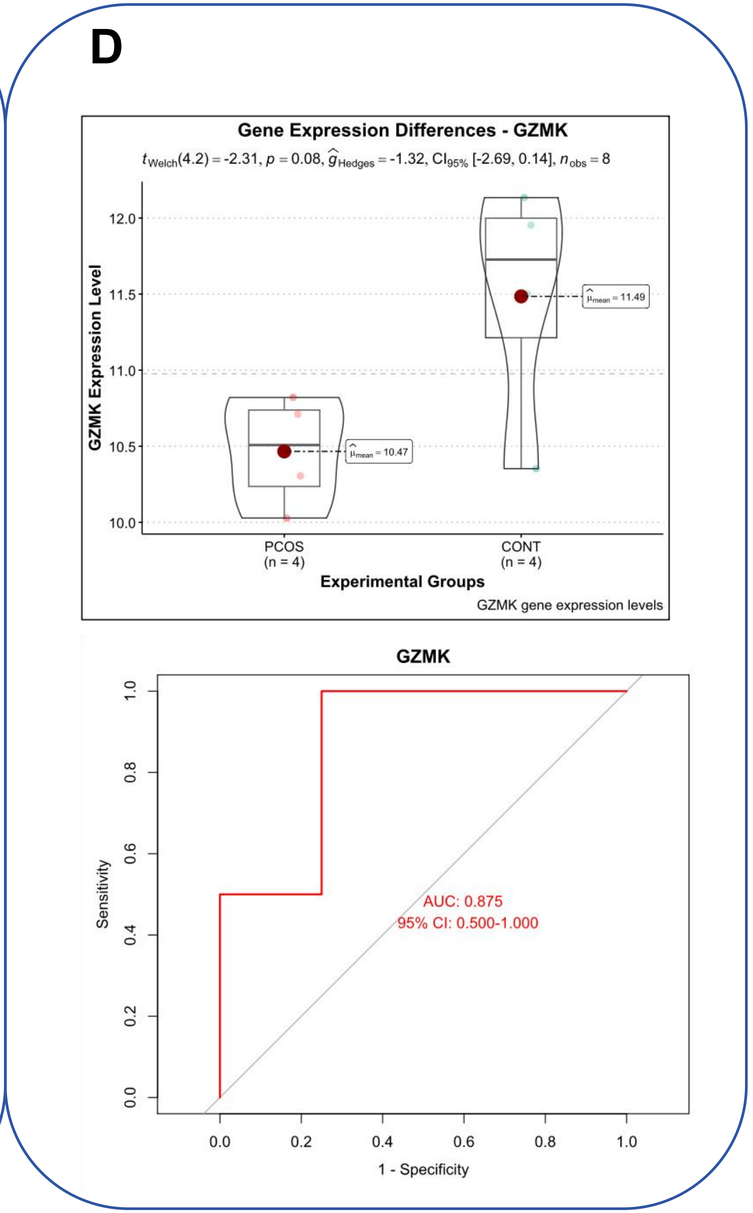
D
Gene Expression Differences - GZMK
$t_{Welch}(4.2) = -2.31$, $p = 0.08$, $\hat{g}_{Hedges} = -1.32$, $CI_{95\%}$ [-2.69, 0.14], $n_{obs} = 8$
GZMK Expression Level
$\hat{\mu}_{mean} = 10.47$
$\hat{\mu}_{mean} = 11.49$
PCOS (n = 4)
CONT (n = 4)
Experimental Groups
GZMK gene expression levels
GZMK
Sensitivity
1 - Specificity
AUC: 0.875
95% CI: 0.500-1.000

E

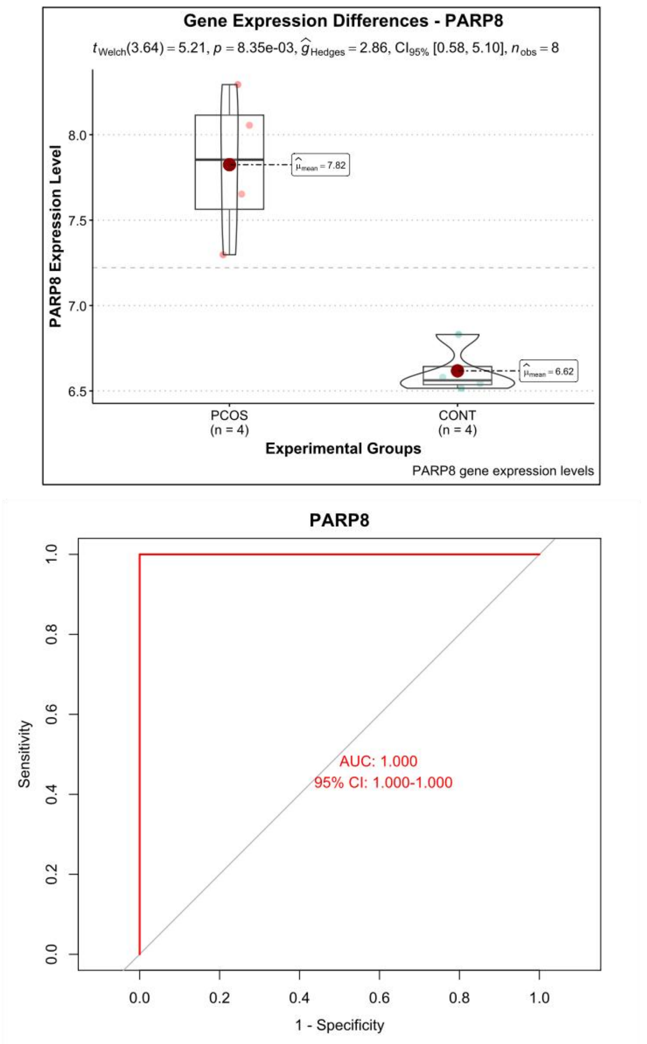
Gene Expression Differences - PARP8
$t_{Welch}(3.64) = 5.21$, $p = 8.35e-03$, $\hat{g}_{Hedges} = 2.86$, $CI_{95\%}$ [0.58, 5.10], $n_{obs} = 8$
PARP8 Expression Level
$\hat{\mu}_{mean} = 7.82$
$\hat{\mu}_{mean} = 6.62$
PCOS (n = 4)
CONT (n = 4)
Experimental Groups
PARP8 gene expression levels
PARP8
Sensitivity
AUC: 1.000
95% CI: 1.000-1.000
1 - Specificity

F

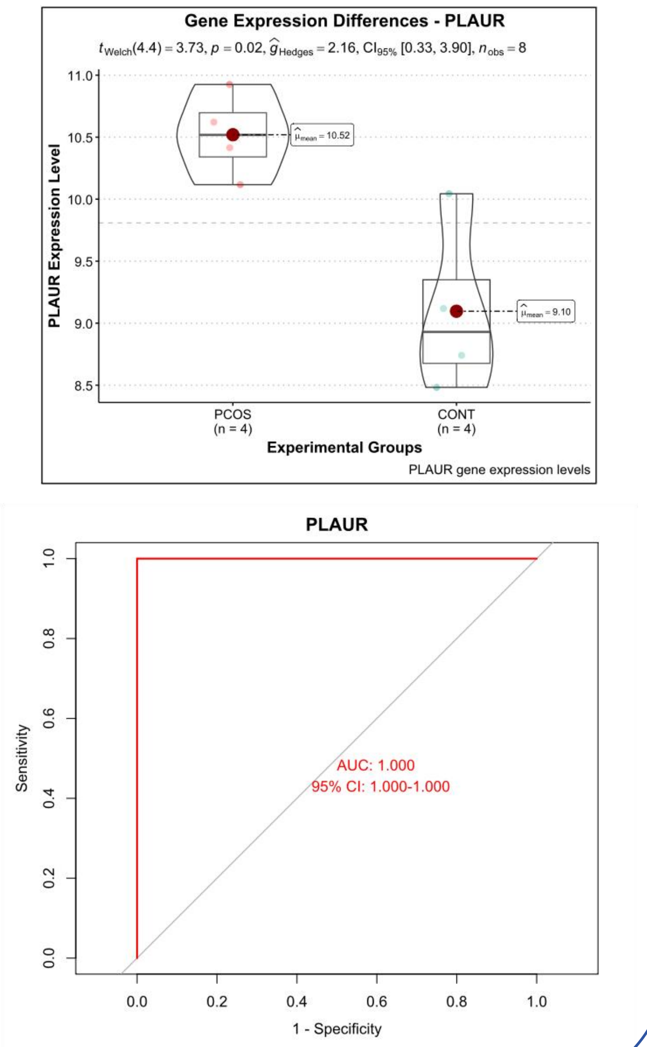
Gene Expression Differences - PLAUR
$t_{Welch}(4.4) = 3.73$, $p = 0.02$, $\hat{g}_{Hedges} = 2.16$, $CI_{95\%}$ [0.33, 3.90], $n_{obs} = 8$
PLAUR Expression Level
$\hat{\mu}_{mean} = 10.52$
$\hat{\mu}_{mean} = 9.10$
PCOS (n = 4)
CONT (n = 4)
Experimental Groups
PLAUR gene expression levels
PLAUR
Sensitivity
AUC: 1.000
95% CI: 1.000-1.000
1 - Specificity

G

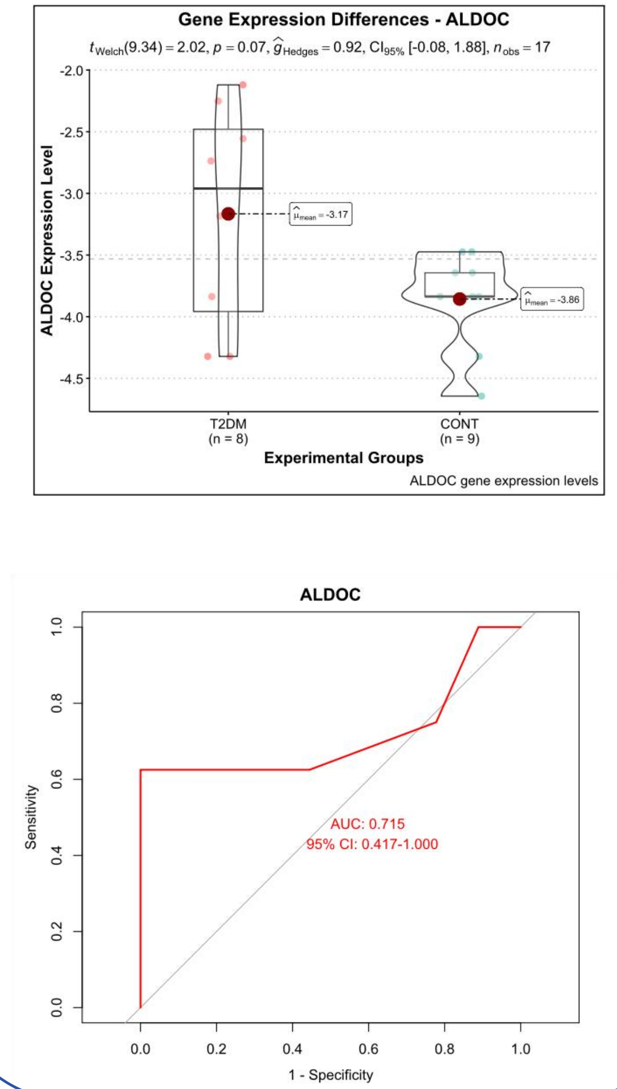

Gene Expression Differences - ALDOC
$t_{Welch}(9.34) = 2.02$, $p = 0.07$, $\widehat{g}_{Hedges} = 0.92$, $CI_{95\%}$ [-0.08, 1.88], $n_{obs} = 17$
ALDOC Expression Level
$\hat{\mu}_{mean} = -3.17$
$\hat{\mu}_{mean} = -3.86$
T2DM (n = 8)
CONT (n = 9)
Experimental Groups
ALDOC gene expression levels
ALDOC
Sensitivity
1 - Specificity
AUC: 0.715
95% CI: 0.417-1.000

H

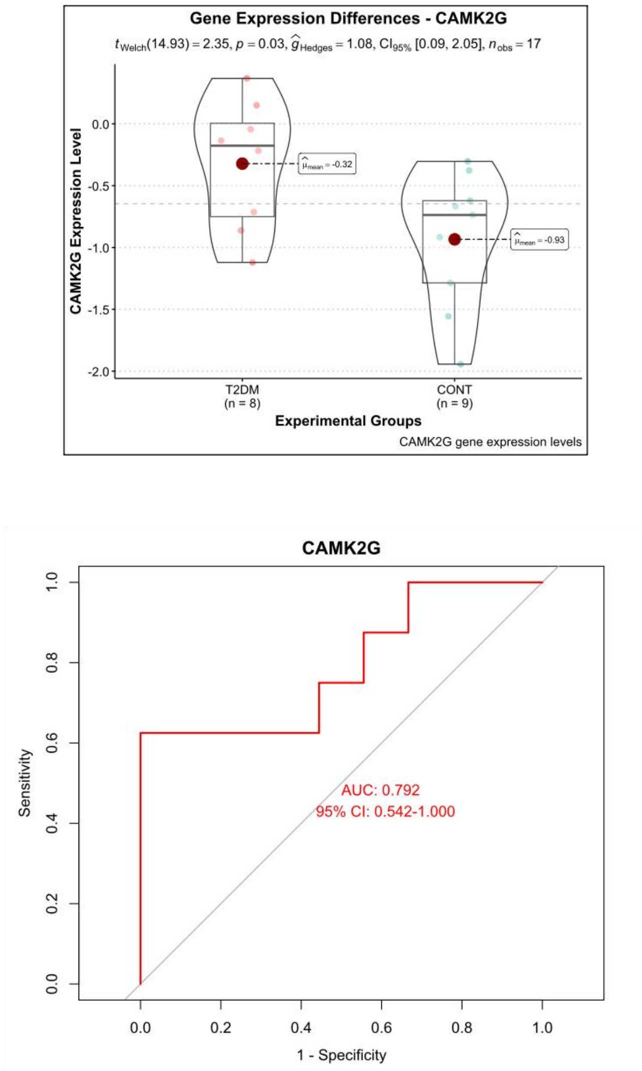

Gene Expression Differences - CAMK2G
$t_{Welch}(14.93) = 2.35$, $p = 0.03$, $\widehat{g}_{Hedges} = 1.08$, $CI_{95\%}$ [0.09, 2.05], $n_{obs} = 17$
CAMK2G Expression Level
$\hat{\mu}_{mean} = -0.32$
$\hat{\mu}_{mean} = -0.93$
T2DM (n = 8)
CONT (n = 9)
Experimental Groups
CAMK2G gene expression levels
CAMK2G
Sensitivity
1 - Specificity
AUC: 0.792
95% CI: 0.542-1.000

I

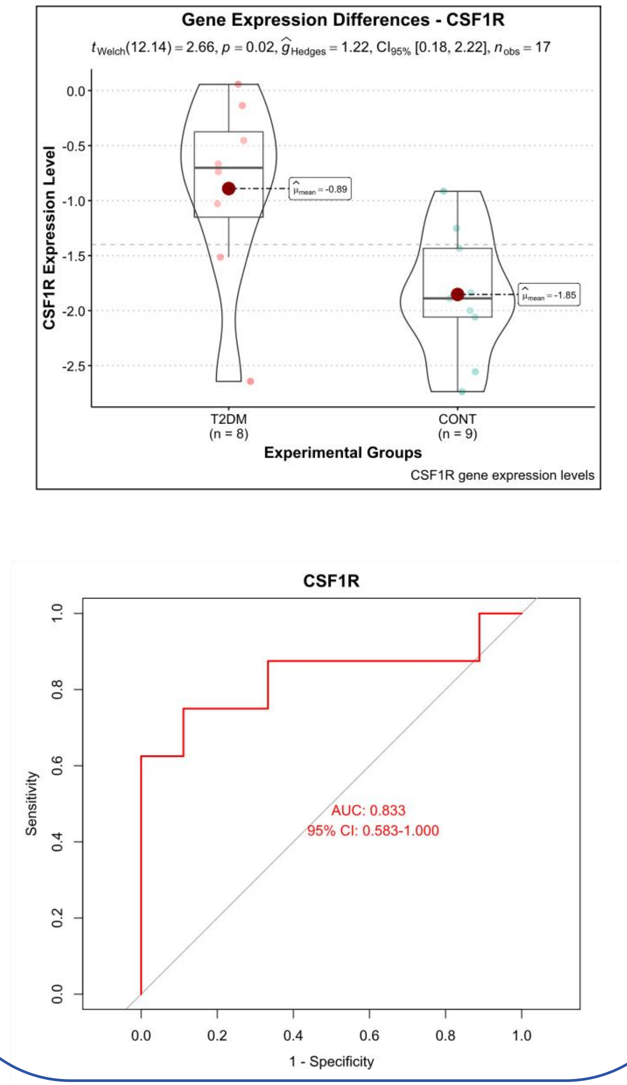
Gene Expression Differences - CSF1R
$t_{Welch}(12.14) = 2.66$, $p = 0.02$, $\hat{g}_{Hedges} = 1.22$, $CI_{95\%}$ [0.18, 2.22], $n_{obs} = 17$
CSF1R Expression Level
$\hat{\mu}_{mean} = -0.89$
$\hat{\mu}_{mean} = -1.85$
T2DM
(n = 8)
CONT
(n = 9)
Experimental Groups
CSF1R gene expression levels
CSF1R
Sensitivity
1 - Specificity
AUC: 0.833
95% CI: 0.583-1.000

J

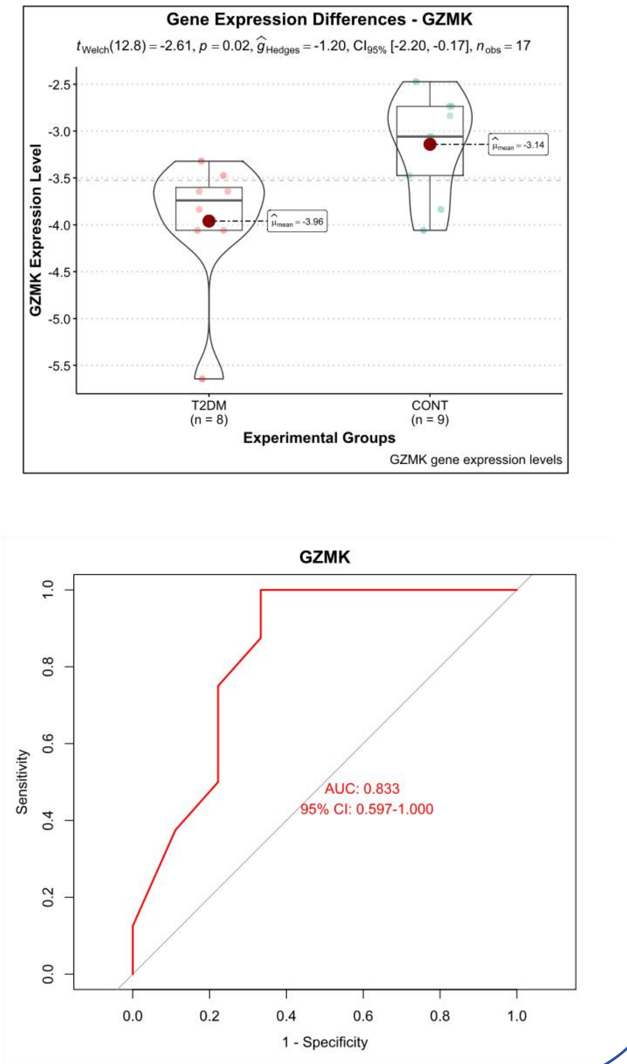
Gene Expression Differences - GZMK
$t_{Welch}(12.8) = -2.61$, $p = 0.02$, $\hat{g}_{Hedges} = -1.20$, $CI_{95\%}$ [-2.20, -0.17], $n_{obs} = 17$
GZMK Expression Level
$\hat{\mu}_{mean} = -3.96$
$\hat{\mu}_{mean} = -3.14$
T2DM
(n = 8)
CONT
(n = 9)
Experimental Groups
GZMK gene expression levels
GZMK
Sensitivity
1 - Specificity
AUC: 0.833
95% CI: 0.597-1.000

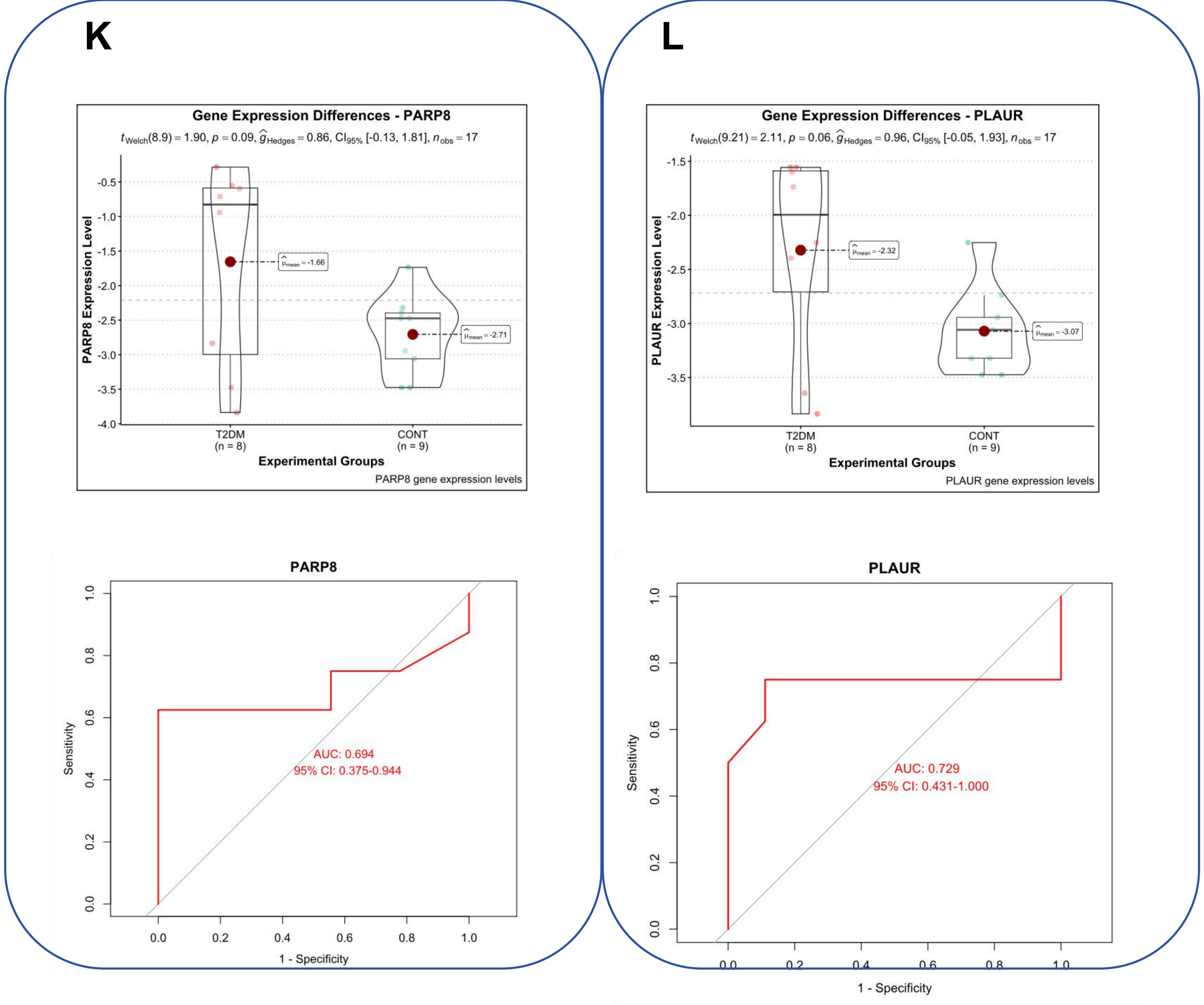
K
Gene Expression Differences - PARP8
$t_{Welch}(8.9) = 1.90$, $p = 0.09$, $\hat{g}_{Hedges} = 0.86$, $CI_{95\%}$ [-0.13, 1.81], $n_{obs} = 17$
PARP8 Expression Level
$\hat{\mu}_{mean} = -1.66$
$\hat{\mu}_{mean} = -2.71$
T2DM (n = 8)
CONT (n = 9)
Experimental Groups
PARP8 gene expression levels
PARP8
Sensitivity
1 - Specificity
AUC: 0.694
95% CI: 0.375-0.944
L
Gene Expression Differences - PLAUR
$t_{Welch}(9.21) = 2.11$, $p = 0.06$, $\hat{g}_{Hedges} = 0.96$, $CI_{95\%}$ [-0.05, 1.93], $n_{obs} = 17$
PLAUR Expression Level
$\hat{\mu}_{mean} = -2.32$
$\hat{\mu}_{mean} = -3.07$
T2DM (n = 8)
CONT (n = 9)
Experimental Groups
PLAUR gene expression levels
PLAUR
Sensitivity
1 - Specificity
AUC: 0.729
95% CI: 0.431-1.000
