# Supplementary material for: Shared senescence-associated gene networks in PCOS and T2DM: biomarker identification and functional validation
Source: Front Endocrinol (Lausanne). 2025 Sep 25;16:1652178. doi: 10.3389/fendo.2025.1652178 (PMC12507634; doi:10.3389/fendo.2025.1652178)
Supplement: Supplementary Figure 2 — Expression levels and AOC curves of hub genes in PCOS_GC_DATASET and T2DM_PBMC_DATASET. (A–F) Expression levels and AOC curves of hub genes in PCOS_GC_DATASET. (G–L) Expression levels and AOC curves of hub genes in T2DM_PBMC_DATASET. [file Image2.pdf]

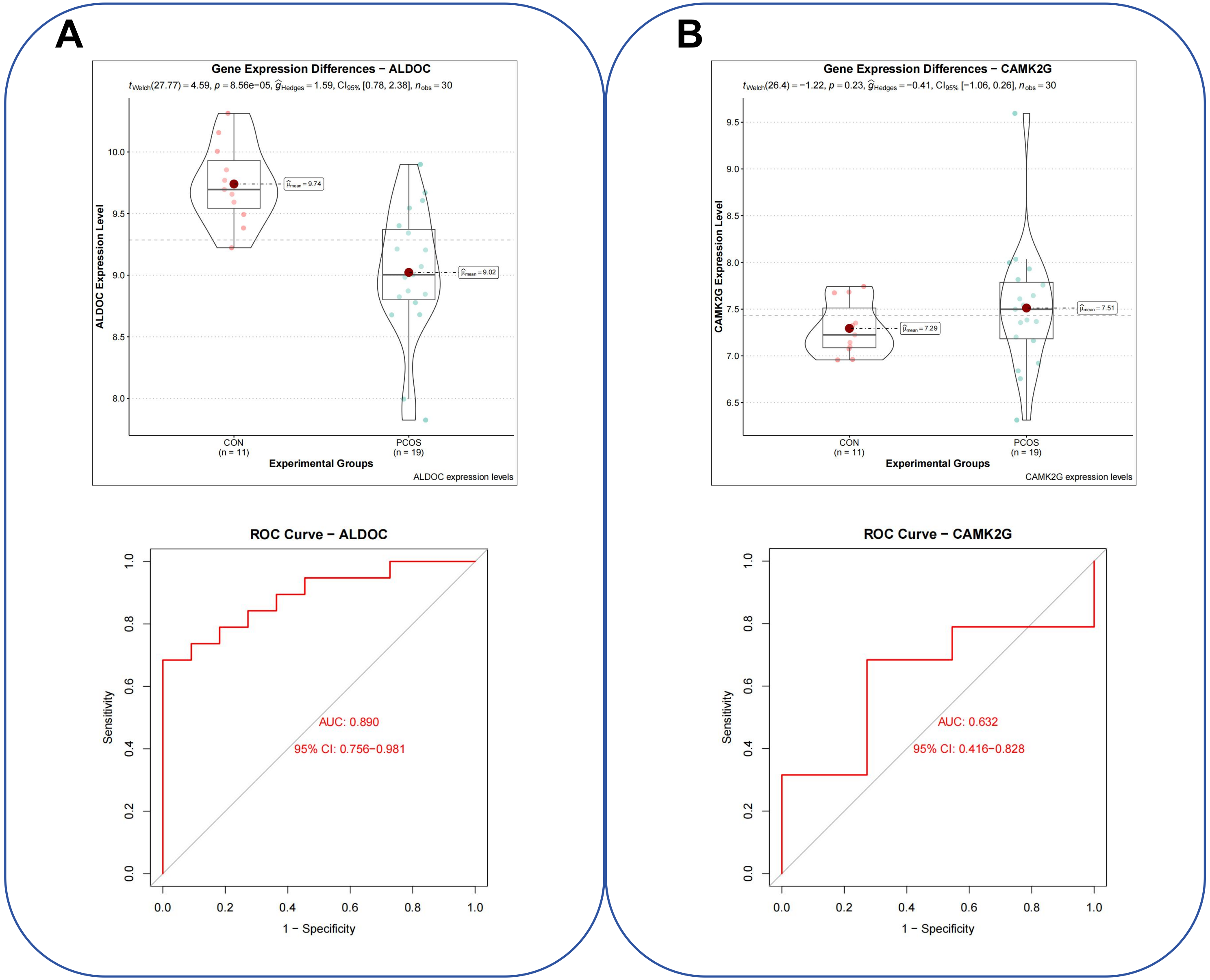

Supplementary Figure 2: Expression levels and AOC curves of hub genes in PCOS_GC_DATASET and T2DM_PBMC_DATASET.
(A-F) Expression levels and AOC curves of hub genes in PCOS_GC_DATASET.
(G-L) Expression levels and AOC curves of hub genes in T2DM_PBMC_DATASET.

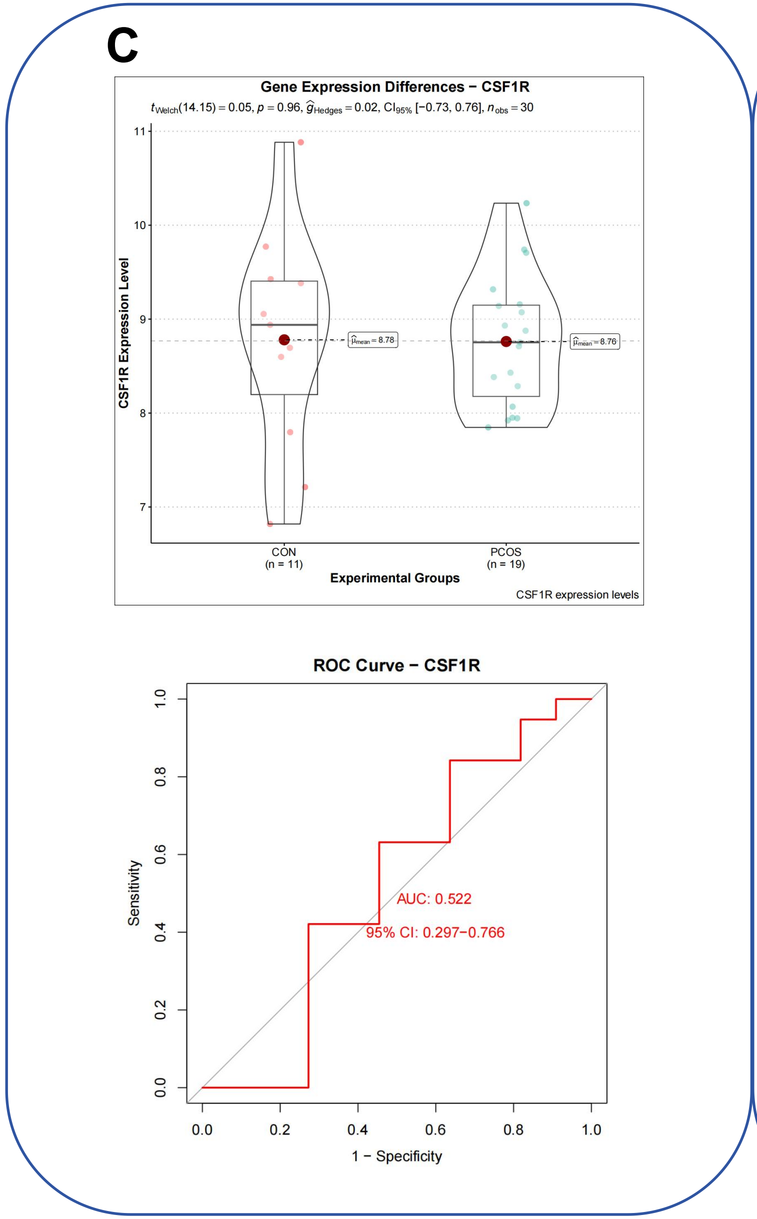
C
Gene Expression Differences − CSF1R
$t_{Welch}(14.15) = 0.05$, $p = 0.96$, $\widehat{g}_{Hedges} = 0.02$, $CI_{95\%}$ [−0.73, 0.76], $n_{obs} = 30$
CSF1R Expression Level
$\hat{\mu}_{mean} = 8.78$
$\hat{\mu}_{mean} = 8.76$
CON
(n = 11)
PCOS
(n = 19)
Experimental Groups
CSF1R expression levels
ROC Curve − CSF1R
Sensitivity
1 − Specificity
AUC: 0.522
95% CI: 0.297−0.766

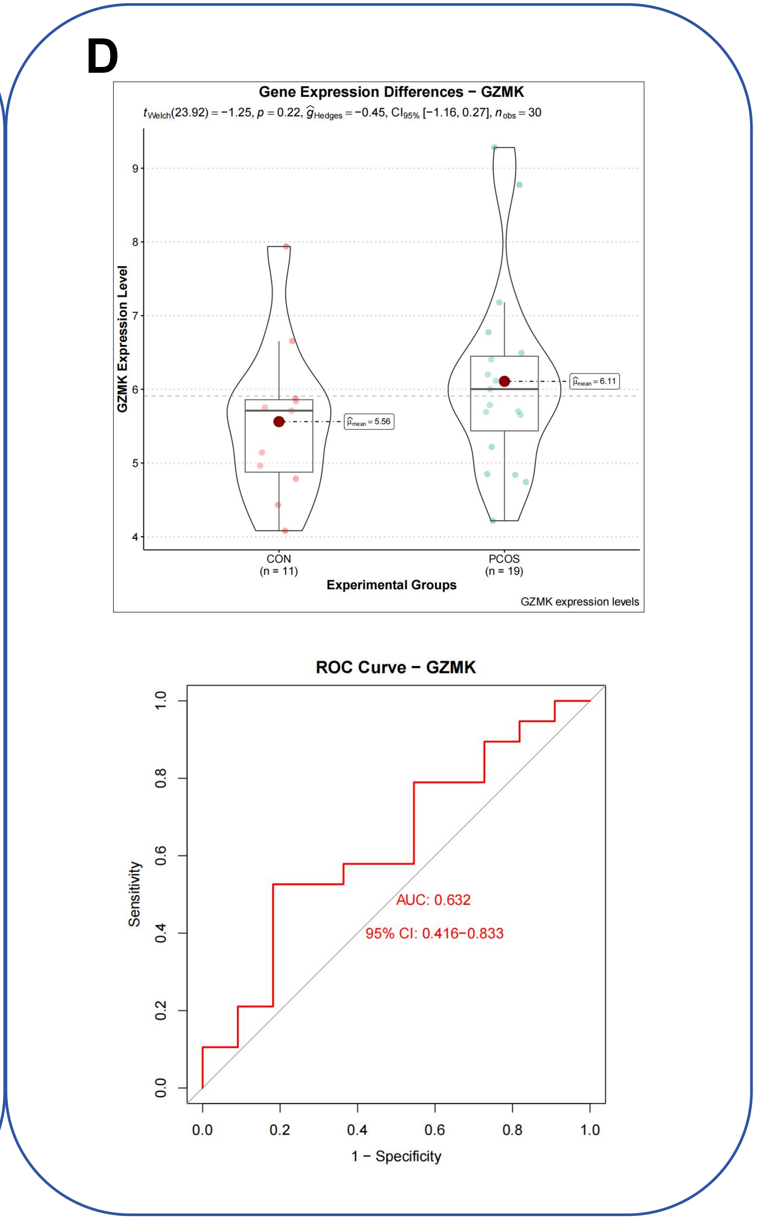
D
Gene Expression Differences − GZMK
$t_{Welch}(23.92) = -1.25$, $p = 0.22$, $\widehat{g}_{Hedges} = -0.45$, $CI_{95\%}$ [−1.16, 0.27], $n_{obs} = 30$
GZMK Expression Level
$\hat{\mu}_{mean} = 5.56$
$\hat{\mu}_{mean} = 6.11$
CON
(n = 11)
PCOS
(n = 19)
Experimental Groups
GZMK expression levels
ROC Curve − GZMK
Sensitivity
1 − Specificity
AUC: 0.632
95% CI: 0.416−0.833

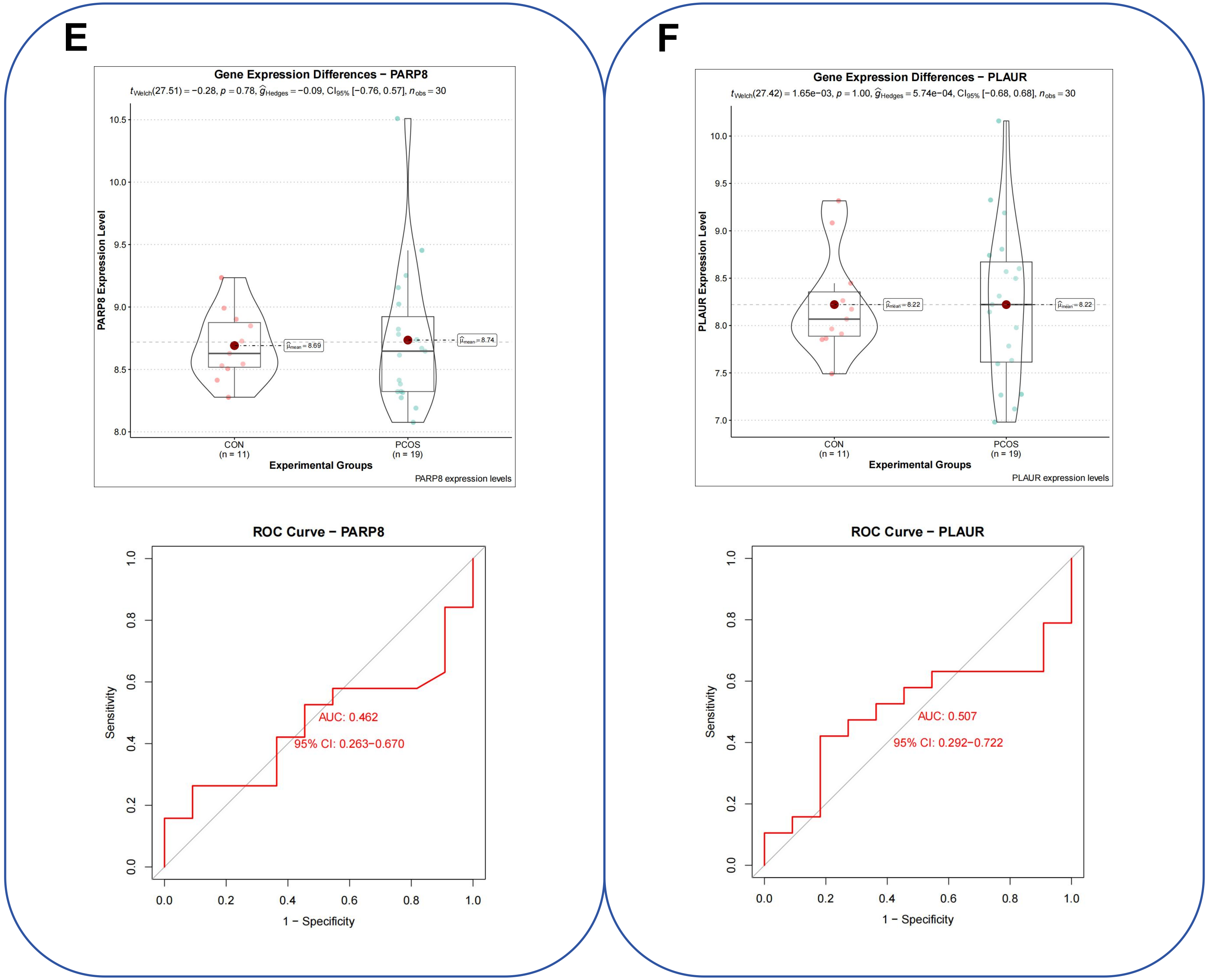
E
Gene Expression Differences − PARP8
$t_{Welch}(27.51) = -0.28$, $p = 0.78$, $\hat{g}_{Hedges} = -0.09$, $CI_{95\%}$ [−0.76, 0.57], $n_{obs} = 30$
PARP8 Expression Level
$\hat{\mu}_{mean} = 8.69$
$\hat{\mu}_{mean} = 8.74$
CON
(n = 11)
PCOS
(n = 19)
Experimental Groups
PARP8 expression levels
ROC Curve − PARP8
Sensitivity
1 − Specificity
AUC: 0.462
95% CI: 0.263−0.670
F
Gene Expression Differences − PLAUR
$t_{Welch}(27.42) = 1.65e-03$, $p = 1.00$, $\hat{g}_{Hedges} = 5.74e-04$, $CI_{95\%}$ [−0.68, 0.68], $n_{obs} = 30$
PLAUR Expression Level
$\hat{\mu}_{mean} = 8.22$
$\hat{\mu}_{mean} = 8.22$
CON
(n = 11)
PCOS
(n = 19)
Experimental Groups
PLAUR expression levels
ROC Curve − PLAUR
Sensitivity
1 − Specificity
AUC: 0.507
95% CI: 0.292−0.722

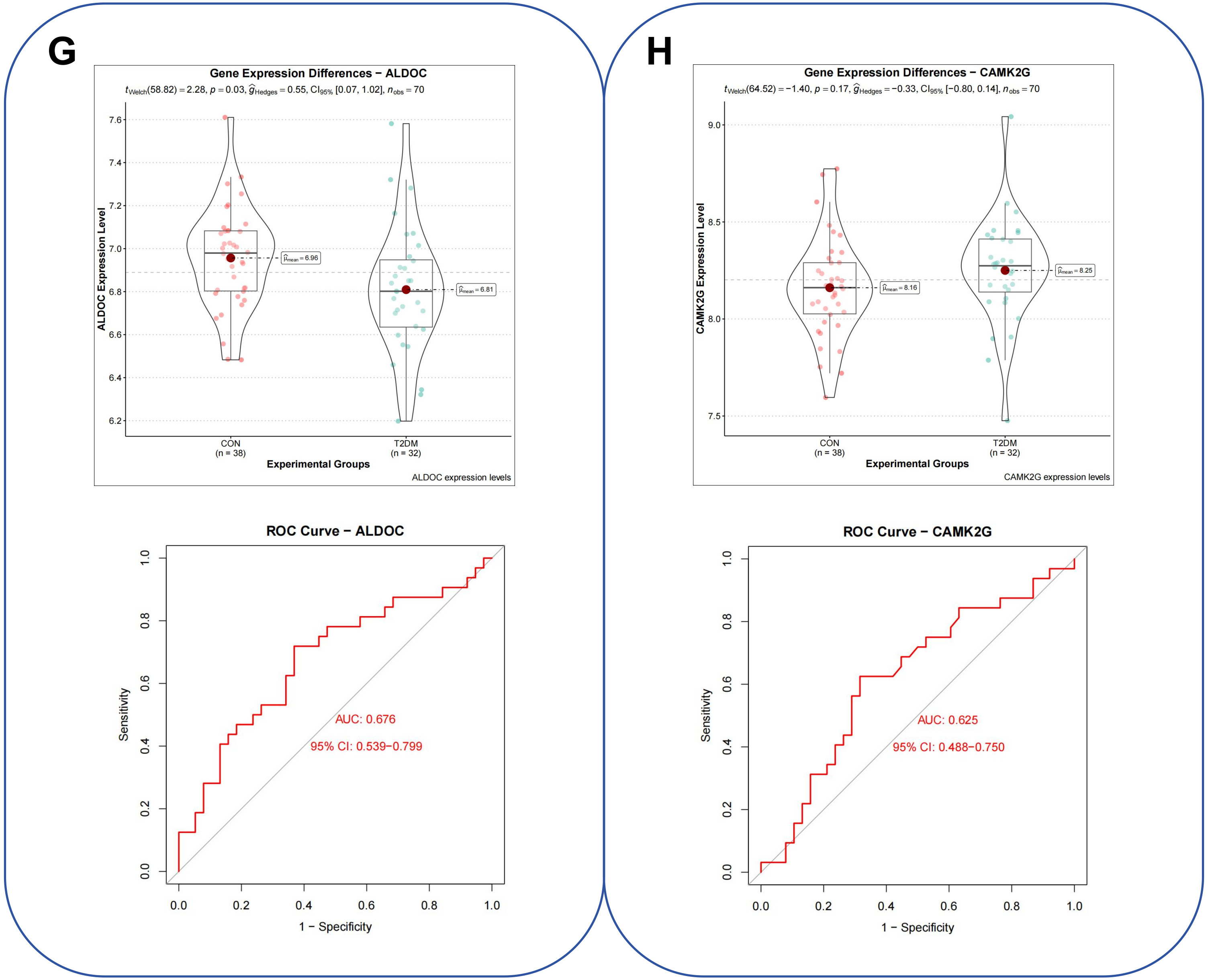
G
Gene Expression Differences − ALDOC
$t_{Welch}(58.82) = 2.28$, $p = 0.03$, $\hat{g}_{Hedges} = 0.55$, $CI_{95\%}$ [0.07, 1.02], $n_{obs} = 70$
ALDOC Expression Level
$\hat{\mu}_{mean} = 6.96$
$\hat{\mu}_{mean} = 6.81$
CON
(n = 38)
T2DM
(n = 32)
Experimental Groups
ALDOC expression levels
ROC Curve − ALDOC
Sensitivity
1 − Specificity
AUC: 0.676
95% CI: 0.539−0.799
H
Gene Expression Differences − CAMK2G
$t_{Welch}(64.52) = -1.40$, $p = 0.17$, $\hat{g}_{Hedges} = -0.33$, $CI_{95\%}$ [−0.80, 0.14], $n_{obs} = 70$
CAMK2G Expression Level
$\hat{\mu}_{mean} = 8.16$
$\hat{\mu}_{mean} = 8.25$
CON
(n = 38)
T2DM
(n = 32)
Experimental Groups
CAMK2G expression levels
ROC Curve − CAMK2G
Sensitivity
1 − Specificity
AUC: 0.625
95% CI: 0.488−0.750

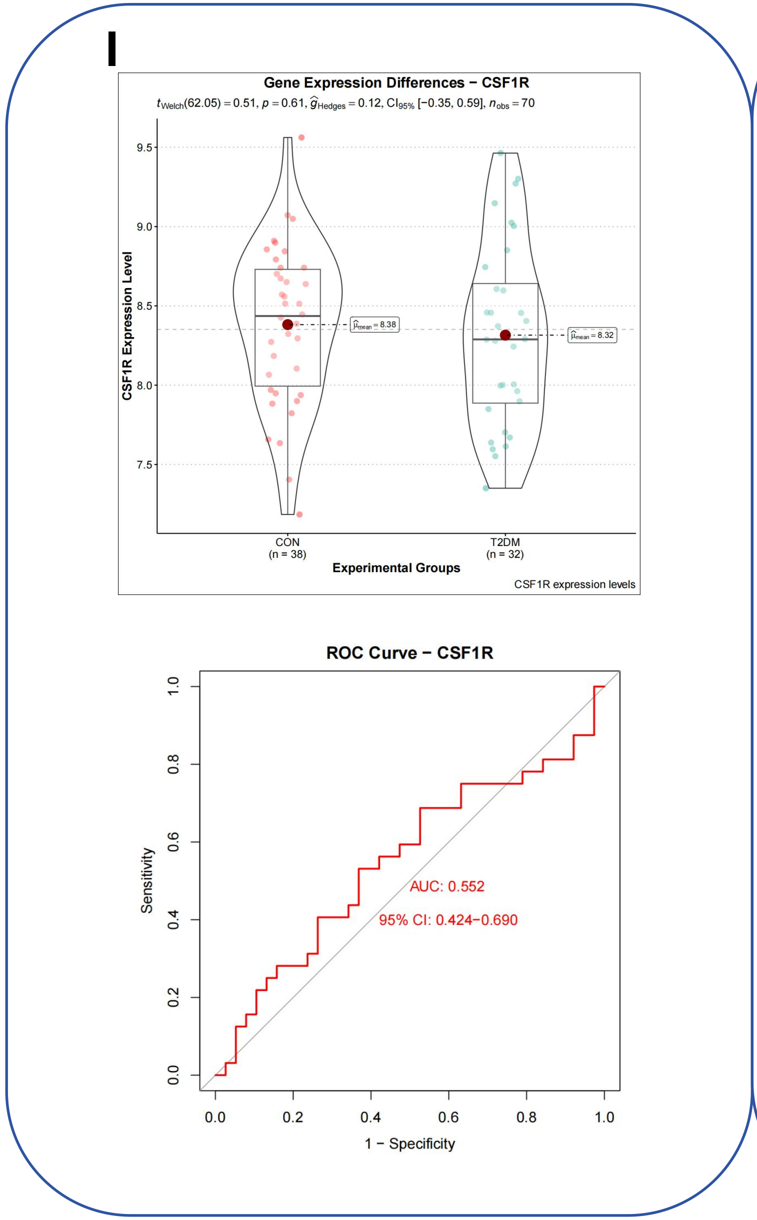
I
Gene Expression Differences − CSF1R
$t_{Welch}(62.05) = 0.51$, $p = 0.61$, $\hat{g}_{Hedges} = 0.12$, $CI_{95\%}$ [−0.35, 0.59], $n_{obs} = 70$
CSF1R Expression Level
$\hat{\mu}_{mean} = 8.38$
$\hat{\mu}_{mean} = 8.32$
CON
(n = 38)
T2DM
(n = 32)
Experimental Groups
CSF1R expression levels
ROC Curve − CSF1R
Sensitivity
1 − Specificity
AUC: 0.552
95% CI: 0.424−0.690

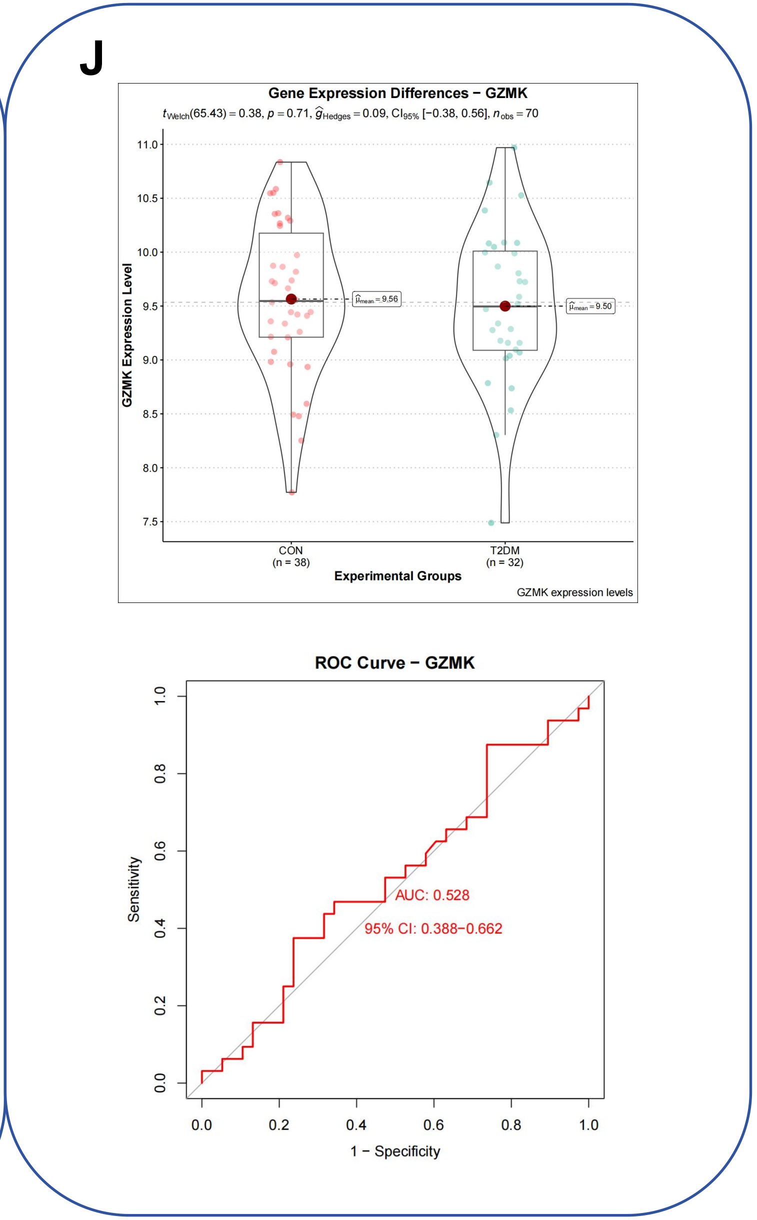
J
Gene Expression Differences − GZMK
$t_{Welch}(65.43) = 0.38$, $p = 0.71$, $\hat{g}_{Hedges} = 0.09$, $CI_{95\%}$ [−0.38, 0.56], $n_{obs} = 70$
GZMK Expression Level
$\hat{\mu}_{mean} = 9.56$
$\hat{\mu}_{mean} = 9.50$
CON
(n = 38)
T2DM
(n = 32)
Experimental Groups
GZMK expression levels
ROC Curve − GZMK
Sensitivity
1 − Specificity
AUC: 0.528
95% CI: 0.388−0.662

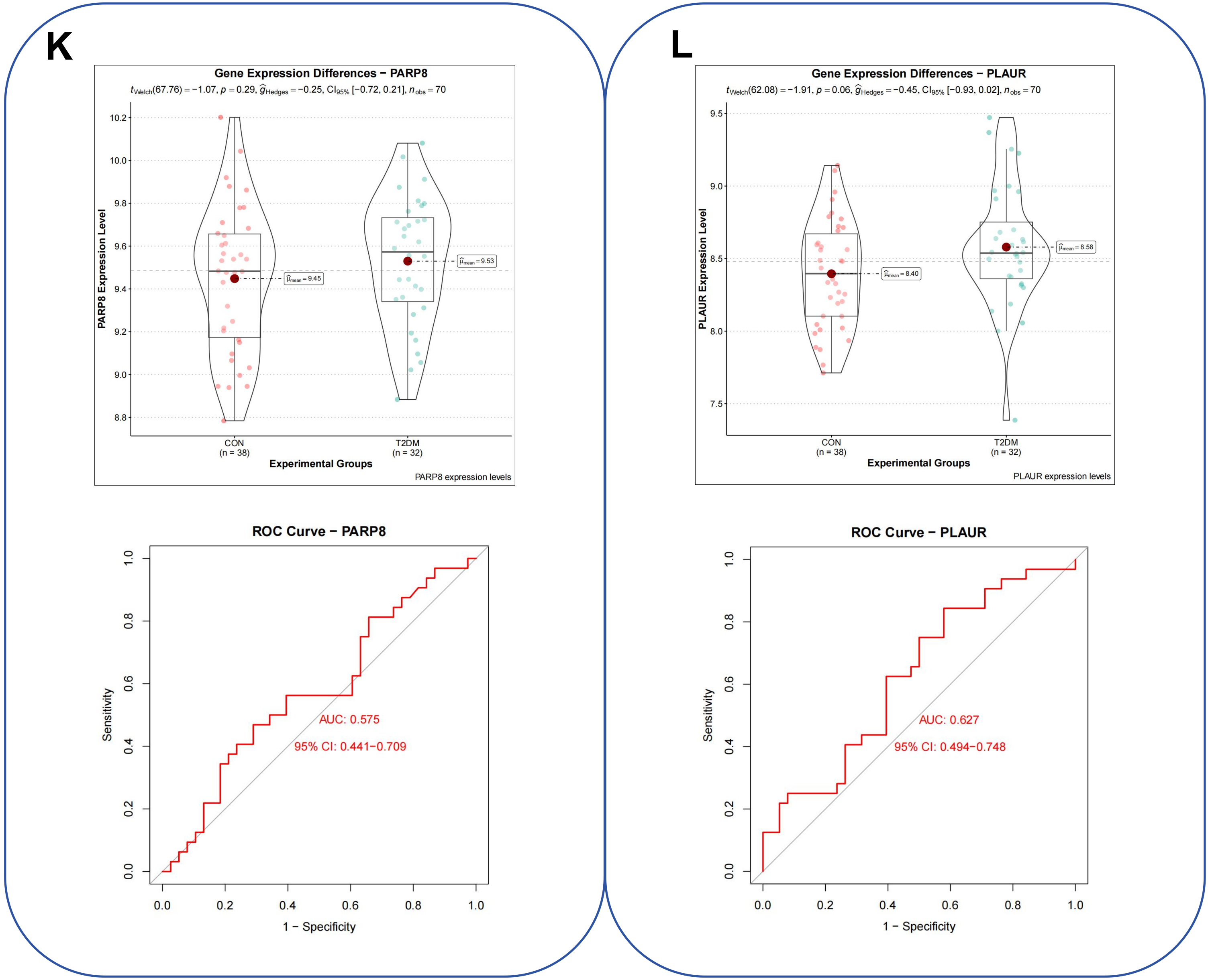
K
Gene Expression Differences − PARP8
t_Welch(67.76) = −1.07, p = 0.29, ĝ_Hedges = −0.25, CI95% [−0.72, 0.21], n_obs = 70
PARP8 Expression Level
μ̂mean = 9.45
μ̂mean = 9.53
CON (n = 38)
T2DM (n = 32)
Experimental Groups
PARP8 expression levels
ROC Curve − PARP8
AUC: 0.575
95% CI: 0.441−0.709
Sensitivity
1 − Specificity
L
Gene Expression Differences − PLAUR
t_Welch(62.08) = −1.91, p = 0.06, ĝ_Hedges = −0.45, CI95% [−0.93, 0.02], n_obs = 70
PLAUR Expression Level
μ̂mean = 8.40
μ̂mean = 8.58
CON (n = 38)
T2DM (n = 32)
Experimental Groups
PLAUR expression levels
ROC Curve − PLAUR
AUC: 0.627
95% CI: 0.494−0.748
Sensitivity
1 − Specificity
